# Dietary Iodine Sufficiency and Moderate Insufficiency in the Lactating Mother and Nursing Infant: A Computational Perspective

**DOI:** 10.1371/journal.pone.0149300

**Published:** 2016-03-01

**Authors:** W. Fisher, Jian Wang, Nysia I. George, Jeffery M. Gearhart, Eva D. McLanahan

**Affiliations:** 1 US FDA, National Center for Toxicological Research, 3900 NCTR Rd, Jefferson, Arkansas, 72079, United States of America; 2 US FDA, Office of Clinical Pharmacology, Office of Translational Sciences, Center for Drug Evaluation and Research, Silver Springs, Maryland, 20993, United States of America; 3 The Henry M. Jackson Foundation for the Advancement of Military Medicine, 2729 R Street, Bldg 837, Wright-Patterson AFB, Ohio, 43433, United States of America; 4 Wright State University Boonshoft School of Medicine, Dayton, Ohio, 45435, United States of America; 5 CDC/ATSDR, Division of Community Health Investigations, 4770 Buford HWY NE, Atlanta, Georgia, 30341, United States of America; University Claude Bernard Lyon 1, FRANCE

## Abstract

The Institute of Medicine recommends that lactating women ingest 290 μg iodide/d and a nursing infant, less than two years of age, 110 μg/d. The World Health Organization, United Nations Children’s Fund, and International Council for the Control of Iodine Deficiency Disorders recommend population maternal and infant urinary iodide concentrations ≥ 100 μg/L to ensure iodide sufficiency. For breast milk, researchers have proposed an iodide concentration range of 150–180 μg/L indicates iodide sufficiency for the mother and infant, however no national or international guidelines exist for breast milk iodine concentration. For the first time, a lactating woman and nursing infant biologically based model, from delivery to 90 days postpartum, was constructed to predict maternal and infant urinary iodide concentration, breast milk iodide concentration, the amount of iodide transferred in breast milk to the nursing infant each day and maternal and infant serum thyroid hormone kinetics. The maternal and infant models each consisted of three sub-models, iodide, thyroxine (T4), and triiodothyronine (T3). Using our model to simulate a maternal intake of 290 μg iodide/d, the average daily amount of iodide ingested by the nursing infant, after 4 days of life, gradually increased from 50 to 101 μg/day over 90 days postpartum. The predicted average lactating mother and infant urinary iodide concentrations were both in excess of 100 μg/L and the predicted average breast milk iodide concentration, 157 μg/L. The predicted serum thyroid hormones (T4, free T4 (fT4), and T3) in both the nursing infant and lactating mother were indicative of euthyroidism. The model was calibrated using serum thyroid hormone concentrations for lactating women from the United States and was successful in predicting serum T4 and fT4 levels (within a factor of two) for lactating women in other countries. T3 levels were adequately predicted. Infant serum thyroid hormone levels were adequately predicted for most data. For moderate iodide deficient conditions, where dietary iodide intake may range from 50 to 150 μg/d for the lactating mother, the model satisfactorily described the iodide measurements, although with some variation, in urine and breast milk. Predictions of serum thyroid hormones in moderately iodide deficient lactating women (50 μg/d) and nursing infants did not closely agree with mean reported serum thyroid hormone levels, however, predictions were usually within a factor of two. Excellent agreement between prediction and observation was obtained for a recent moderate iodide deficiency study in lactating women. Measurements included iodide levels in urine of infant and mother, iodide in breast milk, and serum thyroid hormone levels in infant and mother. A maternal iodide intake of 50 μg/d resulted in a predicted 29–32% reduction in serum T4 and fT4 in nursing infants, however the reduced serum levels of T4 and fT4 were within most of the published reference intervals for infant. This biologically based model is an important first step at integrating the rapid changes that occur in the thyroid system of the nursing newborn in order to predict adverse outcomes from exposure to thyroid acting chemicals, drugs, radioactive materials or iodine deficiency.

## Introduction

Thyroid hormones are involved in many cellular activities essential to normal body function, including maturation and growth. Elemental iodine is vital for thyroid hormone synthesis within the thyroid gland. Severe iodine deficiency has several adverse health outcomes on growth and development. Pregnant women and fetuses are susceptible to iodine deficiency. An infant with severe iodine deficiency in utero may experience impaired intellectual development, growth retardation, and hypothyroidism [1]. Recent research has linked hypothyroxinemia in pregnant women with detrimental central nervous system effects produced in the fetus [2–6]. Mild iodine deficiency is one of the most common causes of hypothyroxinemia [7]. Thyroid peroxidase antibodies in serum, a marker of autoimmune disease, is found in 5 to 20% of pregnant women and is considered a contributing factor for hypothyroxinemia. Hypothyroxinemia in pregnant women can be biochemically defined as maternal serum free thyroxine (fT4) level below the 10th percentile, with a normal serum thyroid stimulating hormone (TSH) level, which varies somewhat between studies [8]. The incidence rates for maternal hypothyroxinemia during the first and second trimester of pregnancy are 2.1 and 2.3% in the United States [9]. Hypothyroxinemia during the first trimester of pregnancy has been associated with cognitive and psychomotor deficits in children [10].

In the healthy euthyroid nursing or bottle-fed full term infant, studies demonstrating hypothyroxinemia and central nervous system effects were not located in the published literature. However, for infants born preterm, transient hypothyroxinemia (low levels of both total T4 and fT4 in serum) has been reported [11–13]. The clinical consequences of transient hypothyroxinemia in preterm infants appear equivocal primarily because of complications with concomitant non-thyroidal illness and drug usage [12].

For exclusively breast-fed infants, mammary gland milk is the only source of elemental iodine. The mammary gland is capable of concentrating iodide in breast milk via the sodium iodide symporter (NIS), a membrane bound transport protein, that is under the control of prolactin and other hormones [14]. Semba and Delange [14] and Dorea [15] reviewed reports for low breast milk concentrations of iodide in regions of the world with goiter (13–18 μg/L) compared to those with breast milk iodide concentrations exceeding 100 μg/L of iodide. Breast milk measurements of iodide in the United States are limited. Leung et al. [16] compared three reports of mean or median iodide measurements in breast milk for Boston, MA (155,178 and 45.5 μg/L) with Texas (34 to 52 μg/L). The range of inter-individual spot breast milk samples can exceed two orders of magnitude.

The Food and Nutrition Board of the Institute of Medicine (IoM) has a Recommended Dietary Allowance (RDA) for dietary iodine intake of 290 μg/d for lactating women [17]. For infants 0 to 6 months of age, the IoM recommended Adequate Intake (AI) is 110 μg/d [17]. However, there is still a dearth of data on the dietary iodine nutrition in infants in the United States [18]. Semba and Delange [14] suggest that full-term infants require 15 μg /kg body weight (BW)/day of dietary iodine. Using the present model’s infant growth equation, this equates to 53, 55, 64, 74, and 84 μg/d of iodine at birth, and for infants 7, 30, 60, and 90 days of age, respectively. Pre-term infants would require twice this amount of iodine (30 μg/kg/d) for a positive iodine balance to accommodate increasing thyroidal iodine stores. To help ensure adequate iodide intake in exclusively breast-fed infants, the lactating woman should consume 200–300 μg/d of iodine [14].

Recently our in silico laboratory has developed computational models to describe the effects of iodine insufficiency on serum thyroid hormone concentrations in the lactating rat [19] and pregnant woman [8]. These models were conceptualized based on the computational research reported by McLanahan et al. [20, 21], who developed hypothalamic-pituitary-thyroid (HPT) axis models to describe hypothyroidism in the adult rat caused by either iodide deficiency or the environmental contaminant perchlorate. For the lactating rat model, laboratory experiments were conducted with pregnant and lactating rats given rat chow containing a range of iodide concentrations, including iodine deficient diets [22]. The biologically based dose response (BBDR) model for the lactating rat was used to quantify decreases in serum thyroxine (T4) and triiodothyronine (T3) for the iodine deficient diets in relation to losses in iodide stores in the thyroid gland. In the pregnant woman, changes in serum thyroid hormones at steady state in a healthy full-term birth fetus and the pregnant mother were predicted as a function of dietary iodine intake and co-exposure to the food and environmental contaminant, perchlorate. The computational analysis by Lumen et al. [8] quantified the changes in potency of perchlorate to block thyroidal uptake of dietary iodide in the pregnant mom and fetus, for various amounts of iodine ingestion.

In this paper, we describe a biologically based model for the lactating woman and nursing infant, employing equations used for the lactating rat and nursing pup and the pregnant woman [8, 19]. The purpose for developing this lactating mother and nursing infant thyroid hormone model was to describe quantitatively thyroid hormone homeostasis for the euthyroid full-term birth infant and lactating mother and then use this model to evaluate moderate dietary iodine insufficiency in the lactating mother and the nursing infant.

## Methods

In the remainder of the paper, the term iodide, if used, replaces the terms, elemental iodine, iodine, and other forms of iodine such as salts of iodide.

The infant portion of the biological model for the lactating mother and nursing infant predicts serum thyroid hormones from near 5–7 days of age to 90 days of age. At birth, a mechanistic understanding of the HPT axis is lacking. Surges in production of thyroid stimulating hormone (TSH) and T4 result in both high serum levels of TSH and T4 after birth. Serum TSH and T4 concentrations decline in the newborn over the first week after birth [23]. Sufficient information was available to predict urinary iodide in the nursing infant from a few days after birth to 90 days of age. Compared to the pregnant mother [8], there is much less serum thyroid hormone concentration data for the lactating mother. Serum thyroid hormone data reported for US mothers on specific days after delivery and up to 90 days postpartum were used to guide model calibration [24]. Iodide measurements in urine and breast milk were available.

Extreme iodide deficiency, accompanied by maternal and infant goiter and hypothyroidism, has been reported. For example, in Germany, an area well known for iodide deficiency, Heidemann and Stubbe [25] described congenital hypothyroidism in newborns and low urinary iodide concentrations in children. Hypothyroidism was not described in this model.

### Euthyroid Model Calibration and Evaluation

#### Lactating Mother Physiology and Iodide

The model compartments for the mother consist of plasma, slowly perfused with blood (eg., muscle), richly perfused with blood (eg., visera), thyroid gland, thyroid plasma, mammary gland plasma, mammary gland fat and mammary gland milk (S1 Table). Some model parameter values were taken from the literature specifically for lactating women. Urinary excretion of iodide was measured in seven lactating women [26] for postpartum weeks 2, 6, and 12. These urinary iodide excretion clearance values (CLurine, L/hr), along with a postpartum day 1 urinary clearance value, taken from the 3^rd^ trimester mother at birth [8], were used as initial values for the lactating mother model and then adjusted to describe urinary iodide data from Leung et al. [16] (S1 Table). The volume of urine excreted each day (Vurine, L/d) was derived from NHANES survey questionnaires for lactating women [27] (S1 Table).

The rates of thyroidal uptake of iodide in these postpartum lactating women [26] at weeks 2, 6, and 12 were determined from thyroidal clearance values reported by the authors. Thyroidal clearance of iodide values were converted to maximum velocity terms for NIS mediated uptake of thyroidal iodide (Vmaxthy, nmol/hr) by dividing the clearance values by the affinity constant, KmNIS (nmol/L) (S2 Table). At parturition, the lactatingmother’s Vmaxthy was set equal to the value used for the 3^rd^ trimester pregnant mother [8]. Organification (the process of formation of precursor thyroid hormones in the thyroid gland) was a second order parameter (KthybindC, /mol/L*hr/kg thyroid weight) used in a similar manner as the adult rat [21] and lactating rat [19]. KthybindC was fitted to predict near maximal concentration of thyroidal iodide stores (CmaxthybindC, nmol/g thyroid weight) based on the thyroid gland weight (kg) (S2 Table). Bi-directional diffusion of iodide between the thyroid gland and plasma (PAthyC, L/hr/kg) was assumed to be minimal to predict very low concentrations of free thyroidal iodide in the thyroid gland (S2 Table).

Two of three compartments of the mammary gland (S1 Table), the mammary plasma and mammary fat, were based on the estimated volume of the glandular and fatty tissues comprising the mammary gland. The breast milk volume was estimated from reports on milk yield (S1 Table). The volume of mammary plasma (VMB, L) was set to 27.6% of the mammary gland (Vmammary, L). The reported breast volume ranges from 0.2 to over 1.0 L [28–30]. A mean value of 0.431 L (Vmammary), determined by Vandeweyer and Hertens [29], was used. The composition of the non-lactating breast can vary because of proportions of the fatty and glandular tissues (7 to 56% volume fatty tissue in women, n = 20) [29]. The glandular tissue of the mammary gland was only used to calculate Vmammary. Ramsay et al. [28], using imaging, estimated that 35 to 37% of the breast was fatty tissue in 21 lactating women one to six months postpartum. Interestingly the proportion of glandular and fat tissues and the size of ducts are not related to milk production. Based on the Ramsay et al. [28] study the fatty tissue volume (VmamfatC) was set to 35% of the volume of the breast (Vmammary) for the lactating woman iodide model. The volume of breast milk was considered to be a constant volume (VMK = 0.369 L). This simplifying assumption is suitable because the synthesis rate of milk (11–58 ml/hr/breast, n = 6 [31]) rapidly replaces the volume of milk suckled by the infant (29 to 33 ml/hr during the first months of life). The residual amount of breast milk remaining in the breast immediately after nursing (mean value of 109 g with high variability [32]) helps ensure an ample supply of milk each nursing. Residual breast milk (109 ml per breast [32]) plus average feeding volume of 84 ml for productive breast and 67 ml for less productive breast [33] equals a total theoretical milk volume of 0.369 L or 0.1845 L per breast. The mammary glands will hold up to 193 ml of breast milk for a productive breast and 164 ml for a less productive breast [33]. Nursing frequency varies for infants aged one to six months, with nursing frequencies of 11 [33] or 7 to 8 /day [32].

Iodide was assumed to either passively diffuse (bidirectional) into fat stores (PAmamductC, L/hr/kg) of the mammary tissue (very minor pathway) or was transported unidirectional by the NIS protein into breast milk (major pathway) (S2 Table). The PAmamductC value was set to a small value of 0.0001 because of low fat solubility. There were no data located to evaluate iodide kinetics in fat tissue, but it is expected to be low. The NIS in the mammary gland (S2 Table) on the basolateral membrane of alveolar cell mediates transfer of iodide from the plasma into milk. NIS expression is stimulated by hormones oxytocin, prolactin, and estrogens [34]. The maximal velocity for NIS mediated uptake of iodide into the breast milk (VmaxmilkC, nmol/hr/kg) was fit to predict both measured iodide levels in breast milk [35] and infant urinary iodide [16]. No in vitro or in vivo data were found for NIS protein expression in the mammary gland as a function of postpartum days. Partition coefficient values (S2 Table) for the slowly and richly perfused tissue groups, milk, and the thyroid gland were set to values previously used [8, 36].

#### Infant Physiology and Iodide

**Fig 1:**
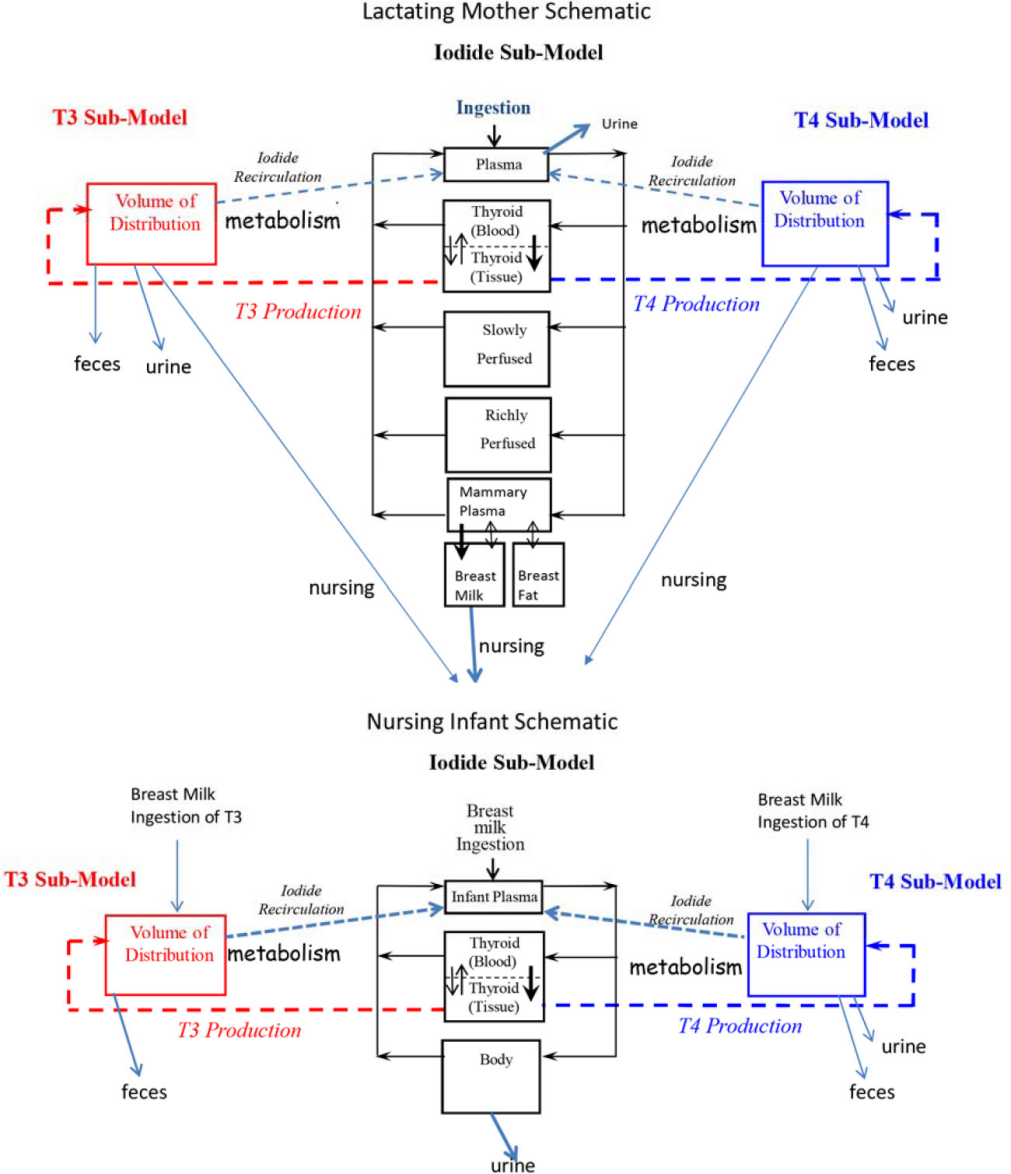
Schematic of the biologically based lactating mother and nursing infant model for thyroid hormone homeostasis from infant birth to 90 days postpartum. The lactating mother iodide sub-model consists of five compartments, two of which are subdivided (thyroid and mammary glands) and in the nursing infant three compartments comprise the iodide sub-model, of which one compartment is subdivided (thyroid gland). The thyroid hormones T4 (thyroxine) and T3 (triiodothyronine) in the lactating mother and the nursing infant are represented using apparent volumes of distribution. Elemental iodine is ingested from food for the lactating mother and from breast milk for the nursing infant. The sodium iodide symporter protein (NIS) actively transports iodide into the thyroid gland of the lactating mother and infant and mother’s breast milk (bold arrows) and is excreted into urine. Thyroid hormones are produced in the thyroid gland and secreted into its volume of distribution (dashed lines). Maternal and infant T4 is metabolized (deiodination, major pathway) to T3 and rT3, and excreted into urine (minor pathway) and breast milk (minor maternal pathway) or conjugated and excreted in feces. Maternal and infant T3 is metabolized (deiodination, major pathway), and excreted in feces and breast milk (minor maternal pathway). Urinary excretion of T3 in the infant and mother, a minor pathway, is not described because of a lack of kinetic data. Iodide released from metabolism of thyroid hormones is recycled into the iodide sub-model and represents an important functional aspect of the HPT axis for maintaining iodide balance. For the infant, important age-dependent functional aspects of the HPT axis are described in the model (see Methods).

The infant iodide model (delivery to 90 days postpartum) was a physiologically based pharmacokinetic model (Fig 1) with the same compartments as the iodide model for the fetus [8]. S1 Table contains infant physiological model parameters for iodide. The infants intake of iodide only occurred by ingestion of breast milk. The nursing rate (Suckle, L/hr) for breast milk was calculated using mean daily milk ingestion rates (NURSEC (g/kg/d) reported by Casey et al. [37] over a 5-day period (S2 Table). Nursing rates for birth, 12 hr, and Days 1, 2, 3, 4, and 5 were 13, 13, 13, 40, 98, 140 and 155 g milk per kg BW of infant per day (g/kg/d). For lactation months 1 and 2, Hofvander et al. [38] reported milk ingestion rates of 154 and 148 g/kg/d and, at lactation month 3, Dewey et al. [32] reported 130 g/kg/d (S2 Table).

Infant body weight growth (BW, kg) was expressed using an algebraic equation to less than 11 months of age [39]. Plasma volume (Vplasma, L) was calculated using information from Brines et al. [40], who estimated plasma volume in four infants, aged 7 to 21 days. Thyroid gland weight changes (VT, kg) for the first 90 days of life were taken from McLanahan et al. [39] and verified using data from Ogiu et al. [41], while thyroid plasma volume (VTB, L) was derived from Lumen et al. [8]. Urine production volume (VurineC, L/hr/kg) reported for the newborn was 5 ml/hr/kg [42] based on 4-hour experimental periods. Cardiac output (QC, L) changes during growth in the infant [43] were converted from whole blood flow to plasma flow by multiplying the whole blood flow rate by 0.578 (red blood cell volume/ whole blood volume). The plasma flow rate to the thyroid gland (QT, L/hr) was set to 1.6% of cardiac output for plasma as was done for previous thyroid models [8, 19, 44].

Urinary excretion of iodide (KurineC, L/hr/kg) increases from birth to 90 days of age [45] (0.74 ml/min/kg from birth to 3 weeks (n = 3) and 1.1 ml/min/kg (n = 4) over the next few months). The maximal velocity for thyroidal uptake of iodide (VmaxthyC, nmol/hr/kg) was derived from thyroidal clearance calculations [45] similar to the lactating mother, but on a kg BW basis. The thyroidal uptake of iodide decreased with age from 2.5 ml/min/kg for the first 3 weeks of life, to 1.1 ml/min/kg for up to 3 months of age. Passive bidirectional diffusion of iodide (PAthyC, L/hr/kg) into and out of the thyroid gland was described using a fitted permeability coefficient value of 0.01 L/hr/kg (≈0.036 L/hr) based on euthyroid iodide stores. The organification of iodide in the thyroid gland was described using a second order rate constant (KthybindC, /nmol/L*hr/kg thyroid weight) fitted to provide thyroidal iodide stores near the maximal value, which increased with age. The maximal organified iodide stored in the thyroid gland of the newborn (n = 13, average 292 μg iodide) was expressed as a concentration, 1716.7 nmol iodide/g thyroid weight (CmaxthybindC, nmol iodide/g thyroid weight), and the amount of thyroid iodide stores was calculated using the volume of the VT. The thyroid stores at birth were measured in 13 newborns from Canada [46]. The thyroid/plasma partition coefficient value (Pthy, 0.15) was set equal to Lumen et al. [8] and the body/plasma partition coefficient (Pbody, 0.21) was an intermediary value between the muscle/plasma partition coefficient (0.18) and the viscera /plasma partition coefficient (0.4) [8].

#### Lactating Mother Thyroid Hormones

The maternal sub-model model structures for T4 and T3 are one compartment (Fig 1), similar to Lumen et al. [8] for the pregnant woman and fetus, and Fisher et al. [19] for the lactating rat and nursing pup. Several, but not all, model parameters for the thyroid hormones in the lactating mother were initially derived from the pregnant woman [8], which were originally obtained from adult studies in men and non-pregnant women and modified for pregnancy. There is a dearth of data related specifically to lactating women and the HPT axis. Deiodinase III production (S3 Table) from metabolism of T4 was set equal to 50%, thus 50% of metabolized T4 was assumed to be converted to rT3 and the remaining 50% to T3, similar to Lumen et al. [8]. rT3 was not tracked in the model, but was assumed to be instantly deiodinated releasing three iodide atoms into systemic circulation within the iodide sub-model. Urinary (CLurineT4C, L/hr/kg) and fecal (CLfecesT4C, L/hr/kg) elimination clearance constants for T4 were fitted to predict less than 2 μg/d in urine or nearly 1% of the T4 formed each day [47] and, for feces, 10–15% excretion as hepatic conjugates [48] (S3 Table). Excretion of T4 in milk (CLmilkT4C, L/hr/kg) was described using a fitted clearance term to predict less than 1 μg/d of T4 [49] transferred to the nursing infant (S3 Table).

With the above model parameters set, the following model parameters found in S3 Table were adjusted as described below. For T4, the maternal volume of distribution (VDCT4, L/kg) was decreased by 50% from the value reported for the pregnant mother [8], metabolism of T4 (KmetT4C, /hr/kg) to T3 and reverse (rT3, inactive form of T4) was increased slightly from the pregnant mother, and the secretion (production) rate of T4 from the thyroid gland (KprodT4C, /hr/kg) was decreased slightly compared to the pregnant mother [8]. These parameters were visually fit serum total T4 concentrations of 16 lactating women reported in NHANES [24]. The concentration of fT4 (FfT4, unitless) was set to a small fraction of total T4, as done in Lumen et al. [8], and then adjusted (visual fit) to better predict the NHANES data [24] by slightly increasing its value compared to the pregnant mother. Predictions of urinary, fecal, and breast milk clearance of T4 was checked to be consistent with targeted amounts presented above.

For T3, maternal loss to feces (CLfecesT3C, L/hr/kg) was targeted to be less than 8% of the T3 formed [50]. Fisher and Oddie [50] reported 10% of radiolabeled T3 was secreted in feces of adults. T3 excretion in urine (ClurineT3C, L/hr/kg) was fit to predict 2.3 μg/d based on an estimated urinary clearance of 1.7 μg/d in adults [47]. Sufficient data were not located on excretion of T3 into urine or milk. Transfer of T3 to breast milk was included in the model because of its moderate lipid solubility. T3 transfer in breast milk (CLmilkT3C, L/hr/kg) was set to be less than 0.1 μg/d. The production rate for T3 (KprodT3C, /hr/kg) was decreased slightly and the T3 metabolic rate (KmetT3C, /hr/kg) increased slightly from previous values reported in Lumen et al. [8]. The T3 volume of distribution for the lactating woman was decreased by 50% from the pregnant woman reported in Lumen et al. [8] to better predict the NHANES 2007–2012 serum levels of T3 for lactating women.

#### Infant Thyroid Hormones

The sub-model model structures for T4 and T3 are one compartment (Fig 1), the same as for the lactating mother. Unlike the lactating mother, more of the thyroid hormone model parameters for T4 were based on the infant and not a surrogate. The thyroid endocrine system in the body of an infant is ‘revved–up’ compared to an adult, with high throughput or turnover in the infant thyroid gland [51]. Consequently, where scientific studies report age dependent model parameter values for the thyroid endocrine system, these were included into the infant model. S3 Table contains infant thyroid model parameters presented below.

T4 secretion (production) from the thyroid gland (KprodT4C, nmol/hr/kg) for the first few days of life in the infant is 0.535 nmol/hr/kg, then decreases over the first month of life to 0.375 nmol/hr/kg, and then remains stable at 0.322 nmol/hr/kg for up to 11 months of age [51]. These data are derived from publications and unpublished data from Corning Nichols Institute Clinical Correlations Division.

The volume of distribution of T4 (VDT4C, L/kg) was set to 0.31 L/kg based on an analysis of 7 children up to 12 months of age, who were injected with radiolabeled thyroxine [52]. Deiodination of T4 was described as clearance term (KmetT4C, L/hr/kg) by visual fitting of serum T4 and fT4 data from Lem et al. [23] using age dependent values of 0.0035 L/hr/kg for the first 10 days of age and 0.0023 L/hr/kg for >10 days to 90 days of age. The reference euthyroid population from Netherlands consisted of 521 health children 0 to 18 years of age, with a gestational age of > 37 weeks [23]. The sample size was 40 for less than one week of age (not depicted in Figure because a mechanistic understanding of thyroid hormone production is lacking the first days after birth), 31 for ages 8 days to one month, and 54 for ages 1–3 months. The fraction of total T4 in serum that is free (fT4) was set to an initial constant value of 0.00096 (n = 21 newborns [53]), then fitted to measured and free T4 in serum [23].

An age dependent factor was incorporated to describe deiodinase III conversion of T4 to rT3. At birth, deiodinase III is present at relatively high levels and decreases with increasing age [54, 55]. Chopra et al. [54] collected serum profiles for rT3 in 18 infants. At birth to 3 days 50% of the metabolized T4 was assumed to be converted to rT3 (and the remainder to T3), 45% on postpartum days 4 through 5, 40% for postpartum days 6 through 30, and 20% for postpartum days 30 to 90. rT3 was not tracked in the model, but three iodide atoms were assumed to be instantly available from deiodination for systemic circulation in the iodide sub-model.

Approximately 8% of T4 was assumed to be secreted into feces in the infant based on adult experimental findings [56] of 10–15%, mentioned previously. For the infant, the maturation of uridine 5’-diphospho-glucuronosyltransferase (UGT) enzymes [57] responsible for conjugation of T4 were assumed to be less than adult values (eg., UGT1A1). No in vivo data were found for the infant to verify this assumption. For urinary excretion of T4 in the infant information was derived from adults. For the infant, we assumed that ≤ 0.3% of T4 was excreted in urine based on 1% urinary excretion observed in adults [47]. No data were found for the infant to verify this assumption.

The volume of distribution for T3 in the infant was set equal to the fetus [8]. The production rate was set equal to 1/11 of the basal T4 production rate [8]. Fisher and Oddie [50] reported that fecal excretion of radiolabeled T3 was about 10% in adults. We targeted 4.5% T3 excretion rate because of the immature status for phase II conjugation [57]. As with T4, T3 metabolic rates were visually fit to predict the mean serum T3 reference interval values reported by Lem et al. [23]. The age dependent parameter values were 0.12 L/hr/kg from birth to 10 days postpartum, and 0.09 for up to 90 days of age.

#### Model Evaluation- Urinary Iodide and Thyroid Hormones

Using the model parameter values found in S1, S2, and S3 Tables to calibrate the model, the following data sets were used to evaluate the model’s ability to predict urinary iodide and serum thyroid hormone concentrations. For the maternal iodide model evaluation of urinary iodide concentrations from 15 lactating mothers were used, who were postpartum on days 1 to 90 and from the United States [24], was used. For model evaluation of the nursing infant, urinary iodide concentrations of infants from the United States, age 1 to 90 days postpartum, were used [18, 58]. Data for model evaluation of infant serum thyroid hormones were taken from Germany [59, 60], New Zealand [61], and Scotland [62]. Verburg et al. [60] reported serum fT4 concentrations (2.5, 50, and 97.7 percentiles) for the infant on days 7, 14, 21, 28, and 90 days of age. Verburg et al. [60] did not report the sample size for each age group. Franklin et al. [61] reported serum T4, fT4, and T3 concentrations on postpartum days 5 (n = 40), 10 (n = 35), and 15(n = 33), and Williams et al. [62] on postpartum days 7 (n = 163), 14 (n = 6), and 28 (n = 9). Elmlinger et al. [59] reported reference intervals (2.5, 50, and 97.5 percentiles) for serum T4, fT4, and T3 concentrations in infants age 1–7 (n = 45) and 8–15 days (n = 40).

### Iodide Deficiency Model Evaluation

The lactating mother and infant studies discussed below represent moderately low intake of iodide over a long duration. Simulations for model evaluation of moderate iodide deficiency were carried out using the model calibrated for the euthyroid lactating mother and infant and simply decreasing the daily intake of iodide. The ability of the model to predict urinary concentrations of iodide in both the lactating mother and infant and breast milk concentrations of iodide were used as a guide to estimate the daily intake of iodide. The model compartments (iodide and thyroid hormones) for the calibrated euthyroid lactating mother and nursing infant were set to starting values at birth (mass for each compartment) determined from simulations in the pregnant mother and term birth fetus [8], assuming an iodide intake of 200 μg/d for iodide sufficiency. For an iodide deficient intake of 150 μg/d the model compartments were set to starting values determined from simulating intake of 200 μg/d in the pregnant mother, and for simulating the lowest dietary iodide intake 50 μg/d the compartment starting values were derived from pregnancy model simulations assuming an iodide intake of 75 μg/d. These approximations for staring compartment values did have some influence on the lactating mother and nursing infant simulations the first few days postpartum.

Costeira et al. [63] reported on iodide status of pregnant women and their children from Portugal. During pregnancy, maternal median urinary iodide concentrations were 57 to 70 μg/L and postpartum, 35–50 μg/L (n = 88 and 105 for maternal postpartum urine samples). These longitudinal low urinary iodide samples suggest dietary intake of iodide was low for this study population. For analyses of iodide, urine, and breast milk, samples were collected from mothers and urine from infants at 3 days and 3 months after delivery. In a later publication, Costeira et al. [64] reported the serum thyroid hormone concentrations (10, 50, and 90 percentiles) for these lactating mothers and nursing infants on days 3 and 3 months postpartum. These data sets were also included in model predictions.

Andersen et al. [65] conducted a cross-sectional study with pregnant women from five cities in Denmark and divided the groups into tobacco smokers and non-smokers. This study occurred before mandatory iodine fortification of salt (1988–1990). On day 5 after delivery, maternal and infant urinary iodide and breast milk iodide were measured in 90 non-smoking mothers and their infants. The mean urinary iodide concentrations were 41 and 50 μg/L for the mothers and infants, indicating moderate iodide deficiency. The mean breast milk iodide concentration was 54 μg/L. TSH and fT4 serum concentrations were reported.

Eltom et al. [66] conducted a longitudinal study with pregnant women from Sudan and followed lactating women for nine months after delivery. Maternal urinary iodide concentrations and serum measurements of TSH and fT4 were reported at 90 days postpartum (number of subjects was not reported, but calculated to be approximately 49). The mean lactating mother urinary iodide concentration was 51 μg/L, indicating moderate iodide deficiency. No infant data were reported.

Hannan et al. [67] reported iodide intake in low-income Mexican-American families from the Rio Grande Valley in south Texas. Breast milk samples were collected on days 30–45 (n = 30) and 75–90 (n = 16) postpartum. Dietary intakes of iodine were estimated to be 76 and 52 μg/day for the second and third months of lactation based on dietary survey information. No infant data or maternal serum TSH or thyroid hormone data were reported. These lactating women were expected to be moderately iodide deficient.

Bouhouch et al. [68] conducted an iodine supplementation study with Swiss mother-infant pairs (n = 241) starting on postpartum weeks 1.6 to 2.8, and whose mean urinary iodide levels were 35 and 73 μg/L for the mothers and infants. The authors also report serum T4 and TSH concentrations in the mothers and infants.

The most data rich, moderate iodine deficient study, for early postpartum maternal-infant pairs was from New Zealand [69]. A randomized, double-blind, placebo-controlled iodide supplementation trial was carried out for lactating women and their nursing infants. Both maternal and infant urinary iodide concentrations were under 51 μg/L in the placebo groups, indicating moderate iodide deficiency. At 6 months postpartum, serum TSH and fT4 were measured and found not to differ between groups.

### Local Sensitivity Analyses

A time-dependent 90 d local sensitivity analysis (SA) was carried out using acslX to assess the impact of a 1% increase in model parameter values in predicting serum fT4 for the lactating mother and for the nursing infant. The local SA was conducted assuming a dietary iodide intake 250 μ/d (iodide sufficiency) or 50 μ/d (iodide deficiency). The normalized sensitivity coefficient (NSC) was calculated using the following equation [70]:

$$NSC=\frac{(O_{i}-O)/O}{(P_{i}-P)/P}$$

where O is the model output resulting from the original parameter value, O_i_ is the model output resulting from the 1% increase in the parameter value, P is the original parameter value, and P_i_ is the parameter value increased by 1%. Parameters with maximum absolute NSC values over 90 days exceeding 0.1 were considered to be sensitive. Manual time dependent (7, 30, 60, and 90 days) local sensitivity analyses were performed for model parameters described with Table functions.

## Results

### Iodide Model Calibration

After exploratory simulations for the euthyroid mother-infant pairs to predict iodide concentrations in both maternal and infant urine, as well in as breast milk, a dietary ingestion rate of 250 μg/d was selected for model calibration purposes as described below. In some of the studies presented below, questionnaires used by investigators asked the volunteers about supplemental vitamin use, some of which contain iodide. An iodide ingestion rate of 400 μg/d is simulated and shown in figures with simulations of the calibrated iodide ingestion rate 250 μg/d, which represents supplemental iodide intake (150 μg/d). The iodide ingestion rates of 250 and 400 μg/d were divided equally among 3 meals over a 12 hr period each day.

Some model parameters were set as described in S1, S2 and S3 Tables for the lactating mother; however, as described in Methods, several maternal model parameters were visually fit in an iterative fashion. The maximal rate for thyroidal uptake of iodide (Vmaxthy, nmol/hr) was set based on Aboul-Khair et al. [26] and decreased from delivery to 90 days postpartum (S2 Table). Organification of iodide in the thyroid gland was visually fit to thyroidal iodide stores using a KthyboundC value of 0.0001 /nm/L/kg thyroid weight coupled with visually fitting a permeability coefficient for iodide and the thyroid gland (PAthyC) equal to a value of 1 x 10^−5^ L/hr/kg (0.0002 L/hr). The maximal rate of uptake of iodide from mammary plasma (across the mammary duct tissue) into milk (VmaxmamC) was visually fit to milk iodide concentrations [35] using a value of 7500 nmol/hr/kg for the first two weeks after delivery, then decreasing linearly to a value of 7000 nmol/hr/kg by 90 days after deliver. The permeability coefficient (PAmamductC) for the mammary tissue was visually fit with a value of 0.001 L/hr/kg. For mammary fat, a permeability coefficient (PAmamfatC) value of 0.0001 L/hr/kg was set for iodide.

**Fig 2:**
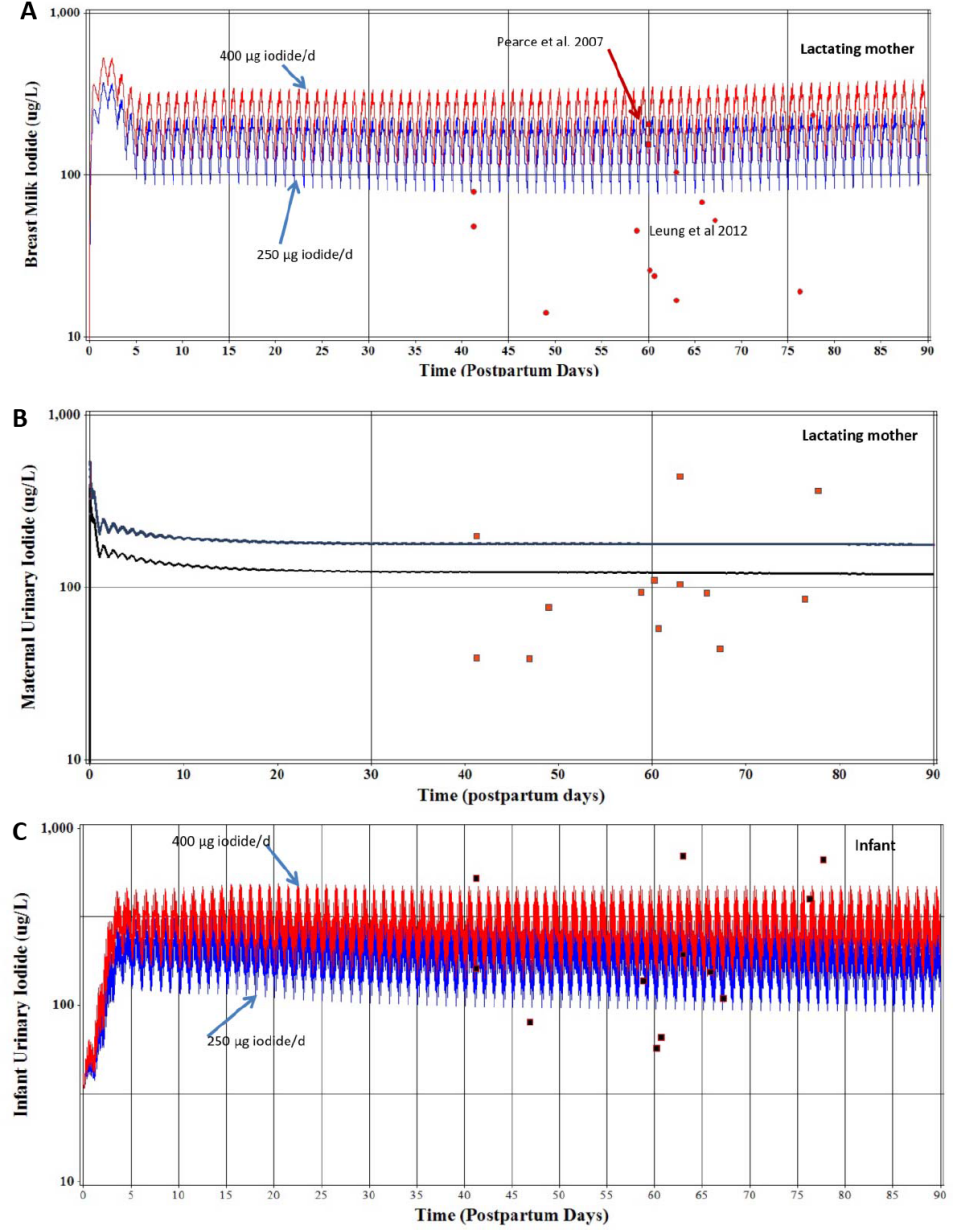
Model calibration predictions are for maternal intake of 250 μg/d iodide, divided equally among three meals per day and an infant nursing eight times during the day. **(A)** Measured concentrations (μg/L) of iodide in breast milk (●) from individual lactating women [16] in Boston, MA USA and reported mean and median values from lactating women (Day 60, ■) representing a wide range of postpartum days [35]. A larger maternal dose of 400 μg/d of iodide is shown representing possible ingestion of supplemental iodine (e.g., vitamins). The daily peak and trough shape of the breast milk concentrations represent the mother’s schedule for daily dietary intake of iodide over a 12 hr period. **(B)** Measured concentrations (μg/L) of maternal urinary iodide (■) from individual lactating women [16] in the Boston, MA USA. Model calibrated prediction of urinary iodide concentration for maternal dietary iodide intake of 250 μg/d with accompanying prediction for 400 μg/d maternal dietary iodide. **(C)** Measured concentrations (μg/L) of iodide in infant urine (■) from individual nursing infants [16] in Boston, MA, USA. Model calibrated prediction of nursing infant urinary iodide concentration for maternal intake of 250 μg/d of iodide and accompanying prediction for 400 μg/d maternal dietary iodide.

Fig 2A depicts data at 60 days postpartum representing mean and median values for breast milk samples reported by Pearce et al. [35] for 57 lactating mothers from the Boston, MA area for a wide range of lactation days. The calibrated visual fit using maternal dietary iodide intake of 250 μg/d successfully predicted the mean and median breast milk iodide concentrations reported by Pearce et al. [35]. An iodide intake of 400 μg/d would also adequately represent the breast milk iodide concentrations of Pearce et al. [35]. Individual iodide measurements of breast milk samples (n = 13) [16] varied over an order of magnitude, with several low measurements of iodide in breast milk. The daily peak and trough simulated iodide concentrations in breast milk represent combined dietary iodide intake schedule for the mother, three ingestions per day over a 12 hr period (Fig 2A).

The lactating mother urinary iodide from Leung et al. [16] was increased slightly from reported urinary clearance rates for iodide in the lactating mother (S2 Table, CLurine, L/hr) to better predict the central tendency for urinary iodide concentrations for maternal intake of 250 μg/d (Fig 2B). The range of measured individual spot urinary iodide concentrations spans 15 fold, well above and below the model predictions of 250 and 400 μg/d for iodide intake.

Predictions of urinary iodide in the infant (Fig 2C) were conducted by first visually fitting the second order rate constant for organification (KthybindC) with a value of 0.1 /nmol/L*hr/kg to predict the near maximal thyroidal iodide stores with a visually fitted permeability coefficient (PAthyC) value of 0.01 L/hr/kg (0.026–0.36 L/hr from birth to 90 days of age). These nursing infants belong to mothers shown in Fig 2A and 2B [16]. Using model parameters derived from the literature and a mother’s iodide intake of 250 μg/d, the peak and trough predictions of infant urinary iodide adequately represent the central tendency of the data (Fig 2C). The simulated peak and trough values for infant urine reflect the nursing schedule for the infant and mother’s eating schedule over a 12 hr period. Urinary iodide concentrations spanned about 15 fold for these 12 nursing infants, with measured urinary iodide above and below the model predictions assuming a maternal intake of 250 or 400 μg/d.

### Iodide Model Evaluation

**Fig 3:**
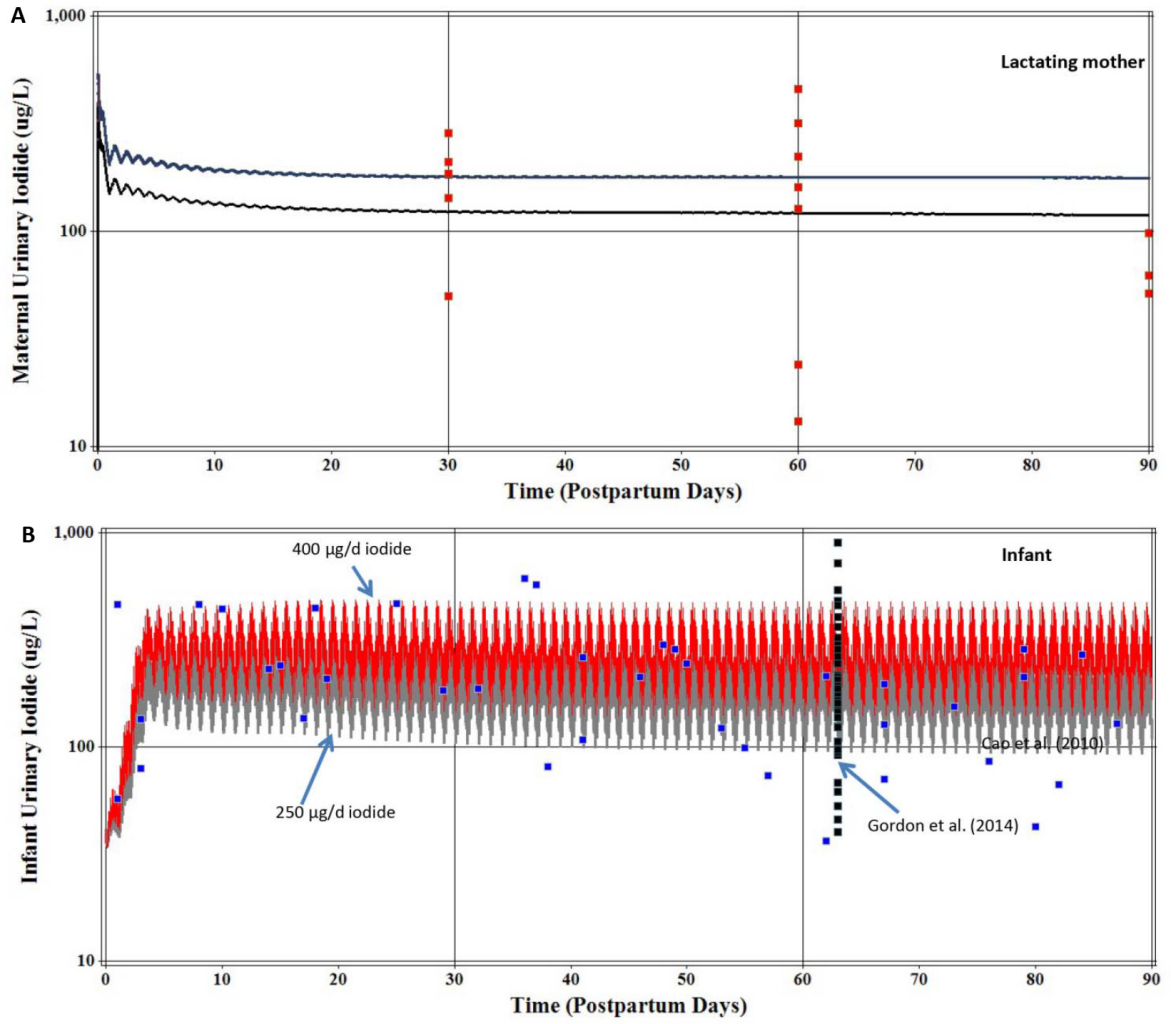
Model evaluation for iodide assuming maternal dietary iodide intake of 250 and 400 μg/d. **(A)** Measured concentrations (μg/L) of iodide in maternal urine (■) from individual lactating women [24] in the United States (250 μg/d, lower line and 400 μg/d, upper line). **(B)** Measured concentrations (μg/L) of urinary iodide from individual nursing infants (■) [58] over 90 days postpartum and another 43 nursing infants [18] shown as an average age of 63 days (■).

Assuming a maternal iodide intake of 250 or 400 μg/d, the model predicted the central tendency of the 15 maternal spot urinary iodide concentrations [24], which ranged 15 fold (Fig 3A). Cao et al. [58] reported infant spot urinary iodide concentrations over 90 days postpartum, which ranged 10 fold (Fig 3B). Assuming an iodide maternal intake of 250 and 400 μg/d, the model provided a reasonable representation of infant urinary iodide concentrations (Fig 3B), with a portion of the data over predicted after 30 days postpartum. Fig 3B shows another study in which infant urinary iodide concentrations are reported for the average infant age of 2.1 months [18]. Maternal iodide intakes of 250 and 400 μg/d resulted in reasonable central tendency estimates of the infant urinary iodide concentrations reported by Gordon et al. [18].

### Thyroid Hormone Model Calibration

The calibration of the iodide sub-models for mother and infant required thyroid hormones. An iterative process was employed to calibrate the lactating mother and nursing infant sub-models for T4 and T3 and iodide.

#### Lactating Mother

**Fig 4:**
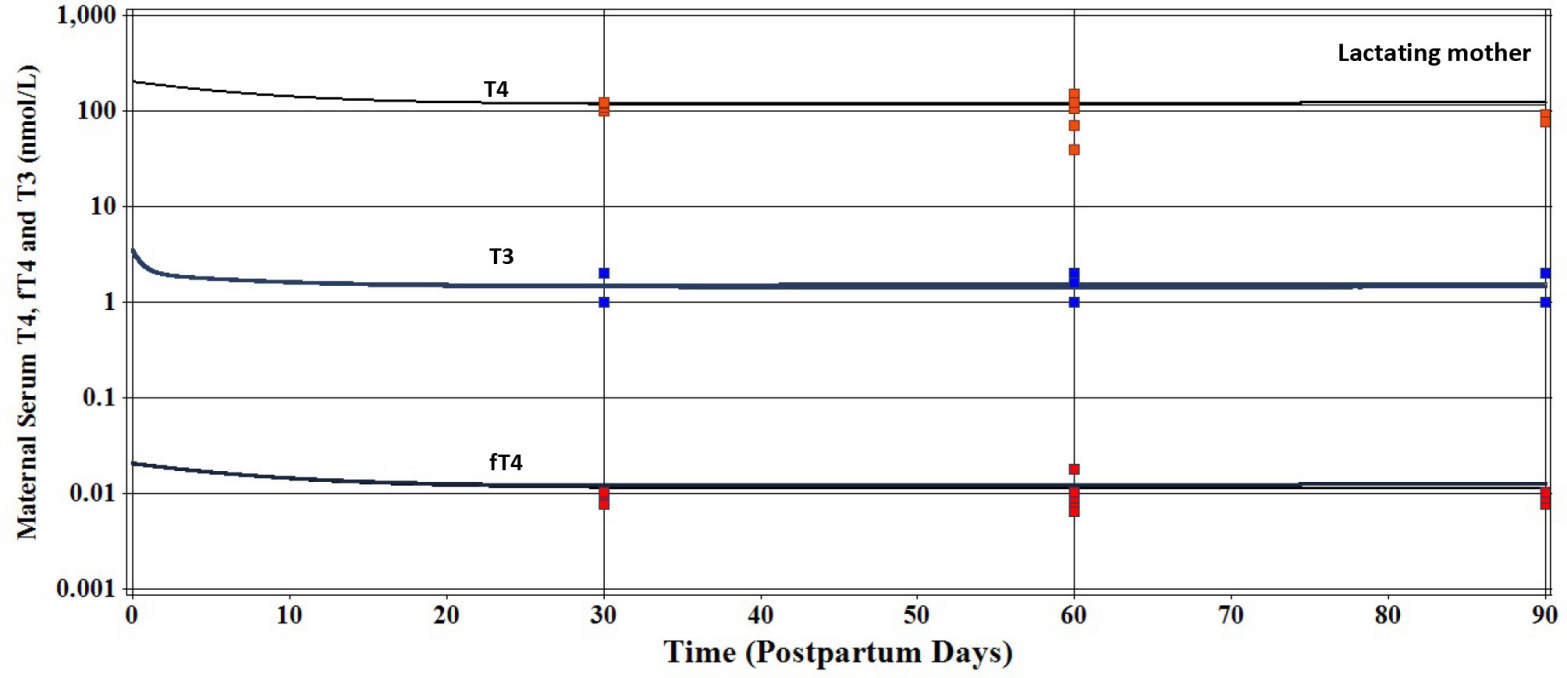
Measured maternal serum T4, fT4, and T3 concentrations (nmol/L) in 16 lactating women (■) residing in the United States [24]. Solid lines represent model calibrated predictions for serum thyroid hormones assuming a maternal iodide intake of 250 μg/d. Simulations performed for maternal dietary intake of 400 μg/d resulted in very slight increases of serum thyroid hormones.

Using the maternal iodide model parameters in S1 and S2 Tables, and ingestion of 250 μg/d of iodide, a refined calibration of maternal serum T4, fT4, and T3 concentrations (S3 Table) was conducted by modifying initial values of the pregnant mother [8] to fit the NHANES 2007–20012 [24] data (Fig 4). Model parameters fitted for T4 included the volume of distribution (VDCT4, 0.09 L/kg), production rate of T4 (KprodT4C, 1.1 x 10^−6^ /hr/kg), and deiodination or metabolism of T4 (KmetT4C, 1.4 x 10^−4^ /hr/kg). The serum fT4 fraction (FfT4, 0.0001 unitless) was also fit to NHANES 2007–2012 data [24]. Model parameters for maternal clearance of T4 in urine (CLurineT4C, L/hr/kg), breast milk (CLmilkT4C, L/hr/kg), and feces (CLfecesT4C, L/hr/kg) were fitted to provide targeted amounts excreted by these pathways based on other adult human studies (S3 Table). Excellent agreement was obtained between model fitted predictions and the limited individual lactating mother serum measurements of T4 and fT4 on postpartum days 30, 60, and 90 (S3 Table). Only a very slight increase in serum T4 and fT4 was discernable when the maternal intake of iodide was increased from 250 to 400 μg/d (Fig 4).

Serum T3 calibration was conducted in a similar manner as T4. The volume of distribution (VDT3C,0.35 L/kg) and metabolism of T3 (KmetT3C, /hr/kg, 2.5 x 10^−3^ /hr/kg) values were fit to NHANES 2007–2012 data (Fig 4). Clearance of T3 into urine (CLurineT3C, L/hr/kg) was described using a value of 0.003 L/hr/kg and fecal elimination of T3 (CLfecesT3C, L/hr/kg) with a value of 0.005 L/hr/kg (S3 Table). Excretion of T3 in milk (CLmilkCT3, L/hr/kg) was described with a value of 2 x 10^−4^ L/hr/kg. Using the fitted model parameters, the maternal serum T3 measurements (Fig 4) were in good agreement with limited available data in lactating women from NHANES [24]. Model predicted serum T3 concentrations were very slightly increased when the dietary iodide intake was increased from 250 or 400 μg/d (Fig 4).

#### Nursing Infant

**Fig 5:**
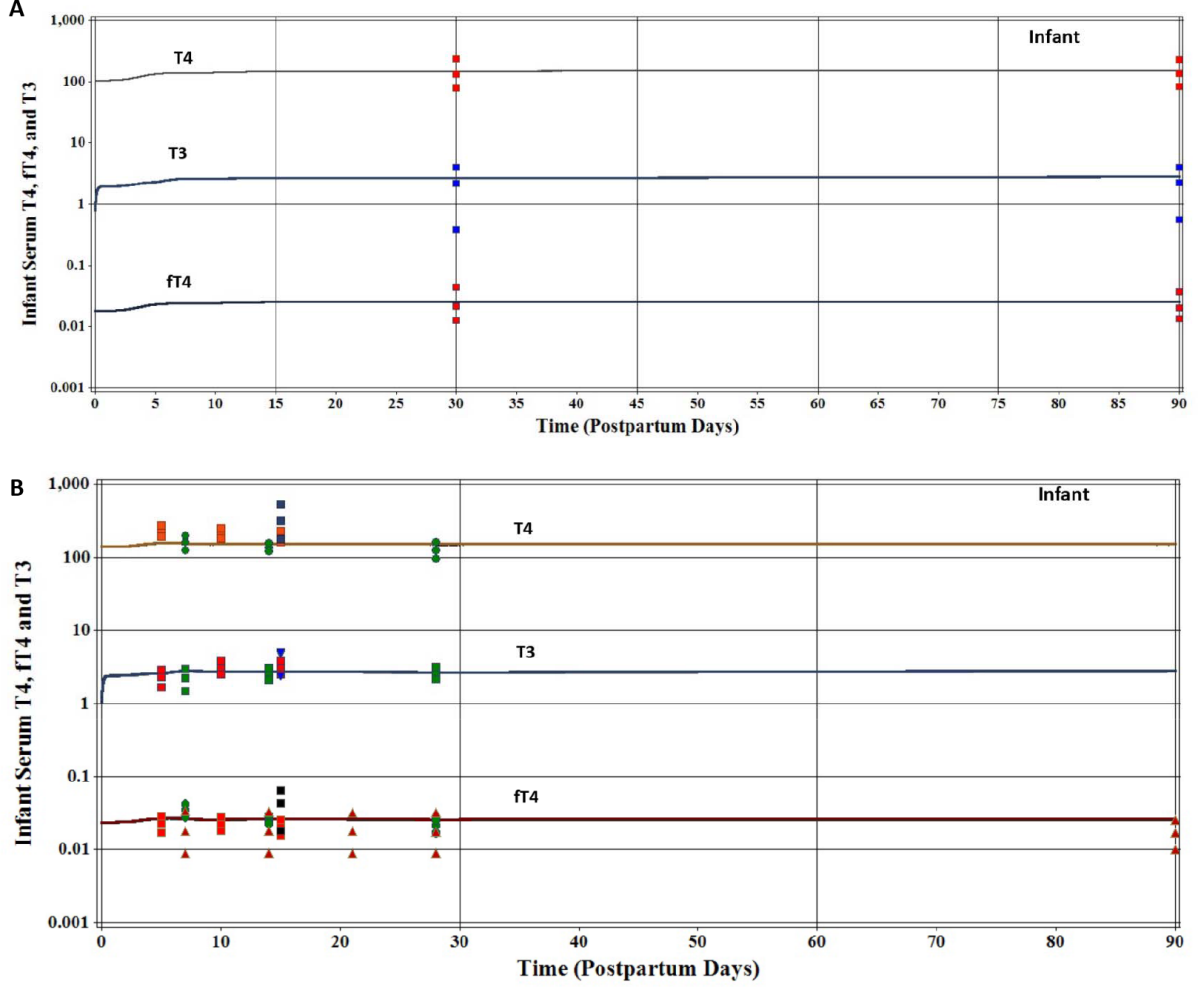
Model calibration (Fig 5A) and model evaluation (Fig 5B) of infant serum thyroid hormones. **(A)** Model calibrated predictions of nursing infant serum thyroid hormones, assuming a maternal dietary iodide intake of 250 μg/d and using reference intervals (2.5, 50, and 97.5%) for infant serum T4, fT4, and T3 concentrations [23]. Infant serum thyroid hormone concentration predictions for a maternal intake of 400 μg/d were identical to a maternal intake of 250 μg/d of iodide. **(B)** Measured and simulated infant serum T4, fT4, and T3 concentrations assuming a maternal intake of 250 or 400 μg/d of iodide. Verberg et al. [60] reported reference intervals (2.5, 50, and 97.5%) for fT4 only in infants from Germany on 7, 14, 21, 28 and 90 days of age (▲). Elmlinger et al. [59] reported reference intervals (2.5, 50, and 97.5%) for 8–15 days of age for infants from Germany and are shown as 15 days of age (■). Franklin et al. [61] from New Zealand reported mean ±SD infant serum T4, fT4, and T3 concentrations on days 5 (n = 40), 10 (n = 35), and 15 (n = 33) (■). Williams et al. [62] from the United Kingdom reported mean ±SD infant serum T4, fT4, and T3 concentrations on days 7 (n = 163), 14 (n = 6), and 28 (n = 9).

For calibration of T4 (S3 Table) several model parameters were set based on literature derived information and maternal ingestion of iodide was assumed to be 250 μg/d. Model parameters that were set included infant volume of distribution of T4 (VDT4C = 0.31 L/kg), age dependent production of T4 in the thyroid gland (KprodT4C = 0.535 nmol/hr/kg at birth and decrease to 0.375 nmol/hr/kg at 90 days of age), urinary (CLurineT4C = 0.0005 L/hr/kg) and fecal (CLfecesT4C = 0.002 L/hr/kg) excretion clearance values for T4 (S3 Table). T4 was calibrated by only fitting the T4 metabolic term KmetT4C (L/hr/kg) in an age-dependent manner to predict the mean values for infant reference intervals for serum T4 and fT4 after 5 days of life to 90 days of age [23]. KmetT4C was set to a value of 0.0035 L/hr/kg for the first 10 days of life, and then lowered to a value of 0.0023 L/hr/kg for the remaining 90 days of life. Reported serum T4 and fT4 reference intervals for umbilical cord serum samples and serum samples collected after birth to 7 days of age were not included in the predictions as explained in Methods, despite good agreement between observation and prediction. The model parameter FfT4 (unitless) was fitted to 0.00017 to predict serum fT4 levels reported by Lem et al. [23]. Fig 5A shows excellent agreement between model predictions of the 50^th^ percentile for serum T4 and fT4 reference concentrations on postpartum days 30 and 90. For T3, the metabolic term KmetT3C was fitted to a value of 0.12 L/hr/kg for the first 10 days of life then decreased to a value of 0.09 L/hr/kg to predict the 50^th^ percentile of the reference intervals for serum T3 (Fig 5A). Infant thyroid hormone predictions for maternal intake of 250 and 400 μg/d were indistinguishable from each other.

### Thyroid Hormone Model Evaluation

#### Lactating Mother

For maternal intake of 250 μg/d of iodide, the model predicted euthyroid serum fT4 concentration in the lactating mother was 0.012 and 0.011 nmol/L at 6 weeks and 3 months after delivery, which were generally below the median or mean values, but near the 1^st^ interquartile values for studies referenced below. Serum fT4 concentrations in lactating women from several countries were reported by Soldin et al. [71]. In Hong Kong, fT4 concentrations were reported for six weeks postpartum (median = 0.0145 nmol/L, interquartile range = 0.0131–0.0160 nmol/L) and 3 months (median = 0.0144, interquartile range = 0.0130–0.0158 nmol/L), at 6 months postpartum in Sudan (median = 0.097, interquartile range = 0.0850–0.0104). Other papers provided mean and reference intervals for serum fT4 levels for lactating women (United Arab Emirates, mean = 0.0137 nmol/L and reference interval = 0.0098–0.0186 nmol/L) or mean fT4 level and standard deviation (Sweden, mean = 0.0137 ± 0.005).

The model predicted euthyroid serum T4 concentration in the lactating mother was 116 nmol/L at 6 weeks and 112 nmol/L 3 months after delivery, which were above the median or mean values for other countries, except for lactating women from Nigeria. Serum total T4 concentrations for lactating women were reported in Hong Kong for 6 weeks postpartum (median = 89 nmol/L, interquartile range = 81–98 nmol/L) and 3 months postpartum (median = 92 nmol/L, interquartile range = 82-101nmol/L). Mean and reference intervals were reported for T4 for lactating women from Belgium (range only = 50–150 nmol/L), Hungry (97 nmol/L, 5–151 nmol/L), Nigeria (140, SD = 35 nmol/L), and Sweden (89.2, SD = 2.9 nmol/L).

The model predicted euthyroid serum T3 concentration in the lactating mother was 1.4 nmol/L at 90 days postpartum, which is similar to the reported values from other countries. Serum total T3 concentrations were reported for 90 days postpartum in lactating women from Sudan (median = 2.2 nmol/L, interquartile range 1.9–2.5 nmol/L), Hungry (mean = 2.0 nmol/L, reference range = 1.2–3.0 nmol/L), Nigeria (mean = 2.5, SD = 0.052 nmol/L), and Sweden (1.9, SD = 0.06 nmol/L) [71].

#### Nursing Infant

With the thyroid hormone model for the nursing infant calibrated using the data set of Lem et al. [23] and maternal intake of 250 μg/d iodide intake, predictions were then compared to other infant thyroid hormone data sets for maternal iodide intake of 250 and 400 μg/d. Refer to figure legend for Fig 5 for details on data sets. Williams et al. [62] serum T4 levels were adequately predicted in infants on days 7, 14, and 28. The model under predicted the mean serum T4 concentrations from Franklin et al. [61] on days 5, 10, and 15 by a factor of 1.3 to 1.4 and for Elmlinger et al. [59] T4 measurements on day 15 by a factor of 2 (Fig 5B). For fT4 and T3, the model predictions were in general agreement with reported fT4 and T3 serum concentrations from Elmlinger et al. [59], Franklin et al. [61], Williams et al. [62], and Verburg et al. [60] (Fig 5B).

### Moderate Iodide Deficiency

**Fig 6:**
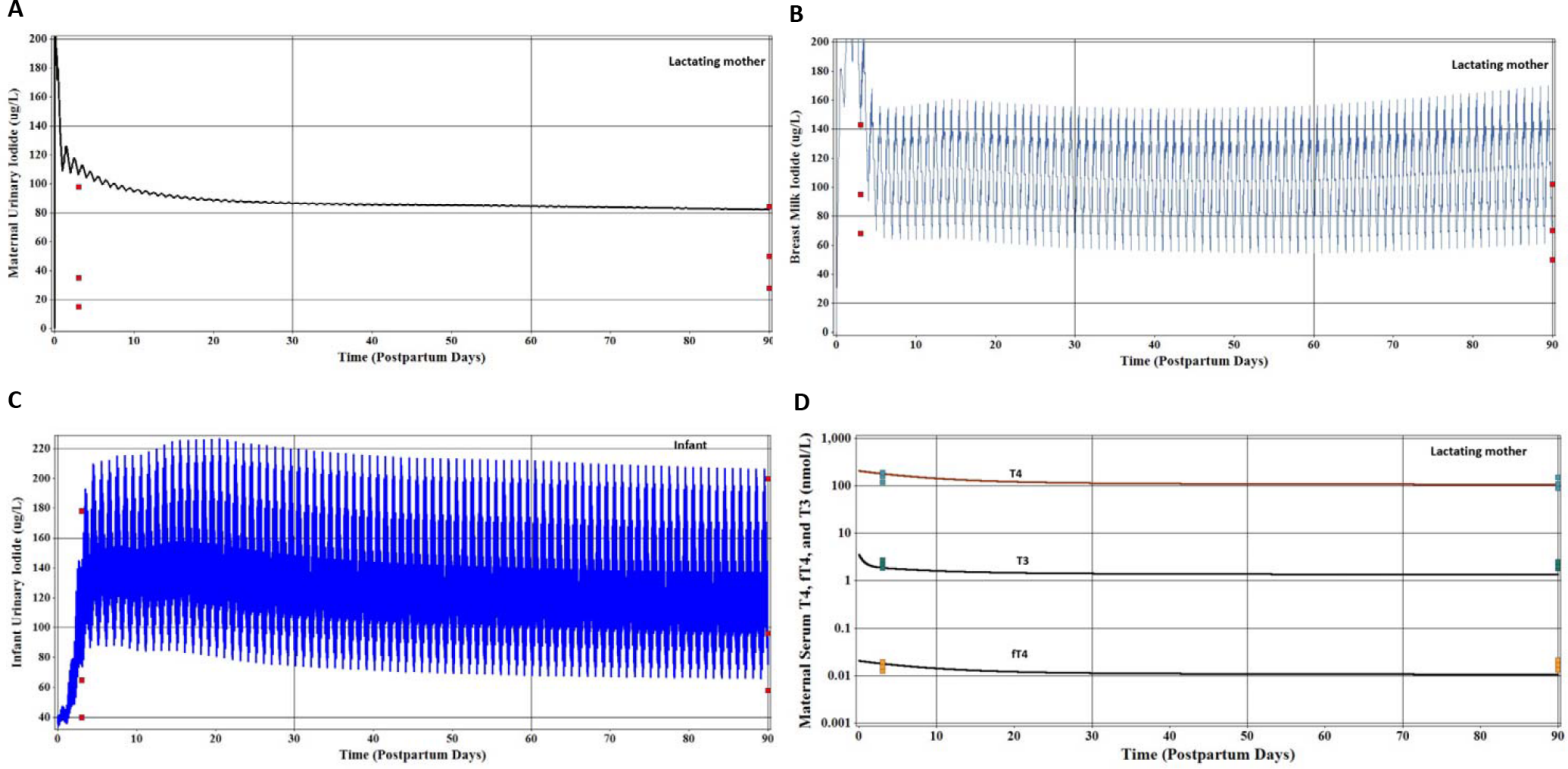
Model predictions of moderate iodide deficiency assuming a maternal dietary iodide intake of 150 μg/d. Costeira et al. [63] measured iodide in breast milk and maternal and infant urine (median and 25 and 75% interquartiles) and later reported maternal serum thyroid hormones [64]. **(A)** Maternal urinary iodide concentrations. **(B)** Breast milk iodide concentrations. **(C)** Nursing infant urinary iodide concentrations. **(D)** Maternal serum thyroid hormone concentrations.

For lactating women-infant pairs from Portugal [63, 64] simulations were performed using the calibrated model for iodide and thyroid hormones for the euthyroid lactating mother and nursing infant. The daily intake of iodide was lowered from 250 to 150 μg/d to seek the best simultaneous agreement between infant and maternal urinary iodide and breast milk iodide concentrations. The simulated maternal urinary iodide concentration (Fig 6A) was above the observed median urinary iodide concentrations by a factor of 2 or greater. For breast milk concentrations of iodide (Fig 6B), general agreement was obtained between observation (25, 50, and 75 percentile) and prediction a few days postpartum and then slightly over predicted breast milk iodide by 90 days postpartum. The peak and trough simulated breast milk iodide concentrations result from mother’s ingestion of iodide (3 times) within a 12 hour period each day.

In the nursing infant good agreement was obtained between observation and prediction (25, 50 and 75 percentile) urinary iodide (Fig 6C). As stated earlier the nursing intervals and the mother’s dietary schedule give rise to the peak and trough infant urinary iodide concentrations. Maternal serum T4, T3, and fT4 were adequately predicted (10, 50, and 90 percentiles) on Day 3 (Fig 6D), despite concerns about possible T4 and TSH surges. At 90 days postpartum there was good agreement between T4 prediction and observation and modest under prediction for T3 and fT4 concentrations (Fig 6D).

Other moderately iodide deficient data sets were simulated by lowering the maternal intake of iodide from 250 to 50 μg/d (S4 Table). Generally speaking, model predictions of urinary iodide and breast milk concentrations were more consistent with experimental findings than serum thyroid hormones (S4 Table), which were predicted within a factor of 2–3. For Sudanese lactating women at 90 days postpartum the model prediction of maternal urinary iodide was within the interquartile values and below the mean reported value. The model predicted serum fT4 and T3, were both lower than experimental findings (S4 Table). The maternal urinary iodide concentration for postpartum day 5 lactating women from Denmark [65] was over predicted, while breast milk and infant urinary iodide concentrations were adequately represented by the peak and trough kinetic behavior of the model. For Danish maternal serum thyroid hormones, both T3 and T4 were under predicted, while serum fT4 was over predicted. For the Danish infants, serum T4 concentration was under predicted, while both fT4 and T3 serum concentrations were over predicted. The model-predicted breast milk peak and trough kinetic behavior of iodide for the Spanish-American population in southwestern United States [67] was in general agreement with the observations (S4 Table) made near the end of 1 and 3 months postpartum. Hannan et al. [67] estimated intake of dietary iodide for this population to be 50–75 μg/day for the lactating mothers. The model-predicted breast milk and the urinary iodide concentrations in the Moroccan lactating mother and nursing infant [68] were in agreement with predictions (S4 Table) on days 10–19 postpartum, assuming an iodide deficient intake of 50 μg/day. Also in agreement with predictions were measured serum T4 levels in the lactating mother and nursing infant (S4 Table).

**Fig 7:**
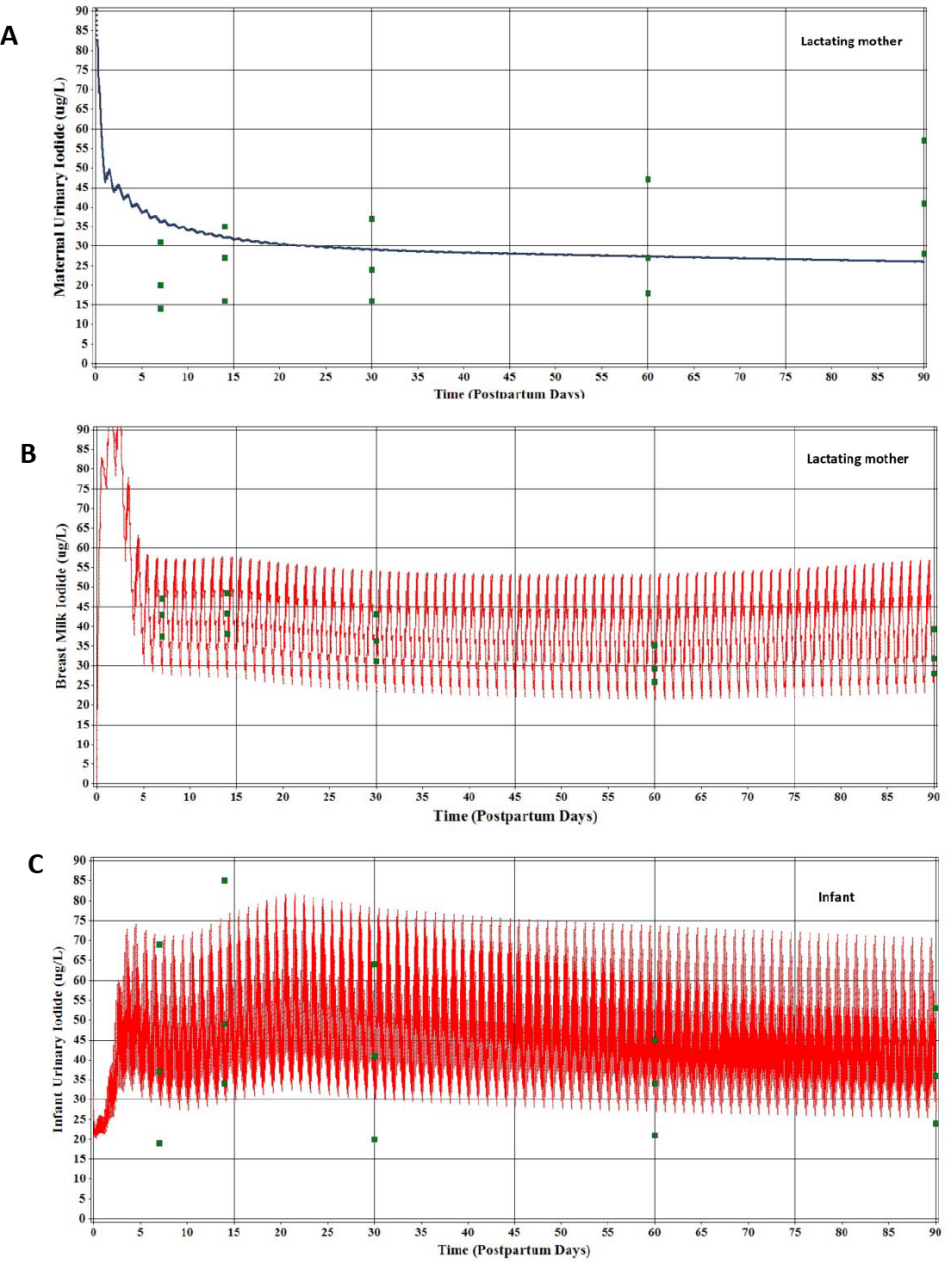
Model predictions of moderate iodide deficiency assuming a dietary iodide intake of 50 μg/d. Mulrine et al. [69] measured iodide in breast milk and maternal and infant urine of an iodide deficient population from New Zealand (mean ±SD). **(A)** Maternal urine. **(B)** Breast milk. **(C)** Infant urine.

For the lactating mother-infant pairs from New Zealand, a maternal iodide intake of 50 μg/d for iodide provided partial agreement between model predictions and observations (mean ±SD) for maternal urinary (Fig 7A). For breast milk iodide concentrations (Fig 7B) and infant urinary iodide concentration (Fig 7C), the peak and trough simulations encompassed the mean and SD for the breast milk and the mean and upper SD for infant urinary iodide.

### Sensitivity Analyses

As expected, the most sensitive parameters (SA >0.5 and <1.1) for the euthyroid and iodide-deficient mother and infant included parameters directly related to fT4 in serum (i.e., metabolism and production of T4 and its volume of distribution, the ratio of fT4 relative to total T4 and body weight (Table 1). The thyroidal iodide stores were very sensitive for the infant in for euthyroid and moderate iodide deficiency, and for the mother, only the euthyroid condition was sensitive. When comparing the moderate iodide deficient mother and infant with each other, more model parameters became sensitive when predicting infant fT4 levels then when predicting fT4 levels in the mother. For the nursing infant, some model parameters were sensitive after several weeks of age and continued to be sensitive to 90 days of age (Table 1).

**Table 1:** Local sensitivity analysis (SA) for serum fT4 in euthyroid and moderate iodide deficient lactating mother and nursing infant. See S1–S3 Tables for model parameter definitions. SA >0.1 and <0.5 ▽, SA>0.5 and <1.1 ▲.

Parameter	Lactating Mom		Nursing Infant	
	Euthyroid	Moderately Iodide Deficient	Euthyroid	Moderately Iodide Deficient
BW	▲	▲	▽	▲
QCC	▽ ▲	▽	▽	▲
QTC	▽▲	▽	▽	▽
VmaxmilkC	▽	▲	▽	▲
KmNIS(milk)	▽▲	▽	▽	
FfT4	▲	▲	▲	▲
KmetT4C	▲	▲	▲	▲
KmNIS(thyroid)	▲			▲
KprodT4C	▲	▲	▲	▲
VDCT4	▲	▲	▽	▽
CmaxthybindC	▲		▲	▲
T3frac				▲
Pthy				▽
VplasmaC				▽
KthybindC			▽	
NURSEC			▽	

## Discussion

A biologically based model was constructed to predict plasma thyroid hormone concentrations in the lactating mother and nursing infant in response to sufficient and moderately insufficient iodide ingestion. To characterize functional aspects of the HPT axis in the model more information was available in the literature for the infant than the lactating woman. The infant HPT axis throughput was very rapid, relative to the lactating mother, with fewer thyroidal stores of thyroid hormones and precursors. To account for the age-dependent changes, Table functions were used (linear interpolation) in many cases by extracting the data from the literature to describe the infant HPT axis. Scaling of these age dependent model parameters based simply on body weight during this rapid growth period was not adequate.

The data sets for iodide in breast milk and urine of the lactating mother and infant were variable, perhaps reflecting several factors such as time since last meal, variable dietary intake of iodide and for urine, and fluid hydration. Model predictions, generally speaking, were adequate for many data sets. Recommendations for sufficient iodide (median concentrations on a population basis) during lactation have been reported for urinary iodide in infants less than two years of age (≥ 100 μg/L [72]), and iodide in urine of lactating women (> 100 μg/L [72]). Researchers have proposed breast milk iodine concentrations of 150–180 μg/L (reviewed in [63]), however no international guidelines exist for breast milk. ICCIDD is now known as the Iodine Global Network (IGN). The model, calibrated with an iodide intake rate of 250 μg/d, provided average iodide concentrations over 90 days postpartum of 161 μg/L for infant urine, 138 μg/L for breast milk and 109 μg/L for maternal urine (excluding the first hour after birth). The nursing infant average daily iodide intake, after 4 days of life, gradually increased from 44 to 88 μg/d by 90 days of life. The IoM RDA for dietary iodine intake for lactating women is 290 μg/day and the AI for infants 0 to 6 months of age, is 110 μg/day [17]. For a maternal intake of 290 μg/d, the model-predicted lactating mother and infant urinary iodide concentrations were 124 and 184 μg/L and for breast milk, 157 μg/L. The nursing infant average daily iodide intake, after 4 days of life, gradually increased from 50 to 101 μg/day over 90 days postpartum.

The American Thyroid Association and the Endocrine Society have recommended that lactating women take vitamins containing 150 μg of iodide daily to supplement their dietary intake of iodide. This recommendation stems from NHANES reports of low individual maternal urinary iodide concentrations in women of childbearing age and pregnant women [73–75]. The NHANES data may be inadequate to draw population-based conclusions from using spot urines for iodide because small sample size [76]. It appears that the expected distribution of urinary iodide spot concentrations from a euthyroid population of lactating women needs to be ascertained before interpreting a small number of individual spot urinary iodide concentration data relative to population iodide sufficiency or insufficiency. The US Food and Drug Administration’s dietary intake study of iodine [77] reported that for women 25 to 30 years, the daily intake of iodide was 148 to 196 μg/d. This provides some baseline information for US women of reproductive age, supporting the recommendations for iodide supplements for pregnant and lactating women.

Model predictions of thyroid hormones in the lactating mother, calibrated using a modest data set reported from NHANES, and compared to other data sets from several countries, was moderately successful, within a factor of two for T4 and fT4, and in agreement with serum T3 levels. These results raise questions about the reproducibility of analytical methods for measuring thyroid hormones in serum [78] and potential differences in serum thyroid hormone profiles across populations. A larger data set for serum thyroid hormones in lactating women from the U.S. is required to calibrate the model for the first 90 days postpartum. For the most part, model parameter values for the lactating mother portion of the model were not derived from lactating women, but from adult humans, both men and women. This provides some uncertainty in model predictions.

The infant model parameters were primarily derived from data in infants, both for iodide and thyroid hormones, including reference intervals for serum thyroid hormones. This was important for the successful description of thyroid hormones in the infant because of the rapid changes that occur in early life. Nevertheless, the calibrated infant model did under predict serum total T4 levels for a few data sets, while acceptable model predictions occurred for infant serum fT4 and T3 concentrations. This was considered an excellent outcome. The variation in measured serum levels of thyroid hormones and iodide in breast milk and urine of mother and the infant demonstrate that a computational description of the HPT axis is a multifaceted problem.

Using the calibrated model to predict iodide concentrations for lactating women who were moderately iodide deficient was successful for an intake of 150 μg/d of iodide [63] and for some of other studies where dietary intake was estimated to be near 50 μg/d for lactating women and nursing infant pairs. In particular, good agreement between prediction and observation was achieved for lactating women and nursing infants from New Zealand [69] and Switzerland [68]. Generally speaking, when the model predictions were not in precise agreement with measurements for serum thyroid hormones, the predictions were within a factor of two of experimental findings. An iodide intake of 50 μg/d for the lactating mother was predicted to result in 31 and 32% reduction in the nursing infant T4 and fT4 serum concentrations, still within the range of reported values for an iodide sufficient infant (Fig 5) and insufficient infant (S4 Table). Because of a lack of an adequate control population to compare to an iodide deficient population of infants, the interpretation of moderate iodide deficiency using biochemical biomarkers for hypothyroxinemia remains unresolved. We also speculate that the different analytical methods used to measure thyroid hormones in serum have a critical impact on the reported serum concentrations of thyroid hormones and thyroid stimulating hormone. Additionally, population differences in homeostatic set points for thyroid hormones, compensatory mechanisms (not accounted for in the model), and uncertainty regarding dietary intake of iodide may contribute uncertainties in the evaluation of moderate iodide deficiency.

Although model parameters were not adjusted, to obtain better agreement between observation and prediction, iodide deficiency may enable non-TSH mechanisms to maintain serum thyroid hormones levels. Vermiglio et al. [79], who were studying iodine deficient lactating women in Italy, speculated that there was enhanced iodide concentration capacity in the mammary gland, perhaps under the control of prolactin. In hypothyroid laboratory rats, the ability to lactate was hypothesized to decrease because of changes in mammary gland nuclear receptors and their co-regulators [80].

Previous investigators have speculated about the importance of lactational transfer of thyroid hormones from mother to infant [49, 81, 82]. That is, maternal thyroid hormones may be an important element for normal thyroid function of the infant. We calibrated our model based on measurements of thyroid hormones in breast milk and estimated that only a few percent of the thyroid hormones are transferred in breast. If this is the case, breast milk is not an important pathway for the nursing infant to obtain thyroid hormones.

The construction of a pharmacodynamic model for thyroid hormones and a kinetic model for iodide require the integration of many published human datasets. While the assimilation of data from diverse areas is a strength of this methodology, it also represents a challenge. There was substantial variability in the reported datasets for iodide concentrations in biological samples. To reduce the uncertainty in model predictions, Monte Carlo methods would help address population differences in both iodide intake and resulting serum, breast milk, and urine iodide concentrations and provide statistical inferences on serum thyroid hormone concentrations as a function of iodide intake. Even with its limitations, this deterministic model provides a quantitative tool to help understand complex interactions of an endocrine system during early development of an infant. The most sensitive model parameter for the euthyroid and iodide deficient infant was thyroidal iodide stores, which was only measured for near birth. More ontogeny information is required to improve the model predictions, perhaps using imaging technology.

### Computer Code

The model code will be provided upon request, written to be executed using acslX simulation language (Version 3.0.2.1) coupled m files containing data sets and other programming information (Aegis Technologies Group, Huntsville, AL). All differential equations have been previously described [8, 19–21], with some minor modifications, therefore equations are not presented in an appendix.

## Supporting Information

S1 Table Mother and Infant (birth to 90 days of age) Physiological Parameter Values and Calculations. (DOC)

S2 Table Mother and Infant (birth to 90 days of age) Iodide Parameter Values and Calculations. (DOC)

S3 Table Mother and Infant (birth to 90 days of age) Thyroid Hormone Parameter Values and Calculations. (DOC)

S4 Table Moderately low maternal dietary iodine intake (50 μg/d) with predicted and observed concentrations of iodide in lactating mother and nursing infant urine and breast milk, and serum thyroid hormone concentrations in lactating mother and nursing infant. Model predictions of serum thyroid hormones were in agreement, less than (↓), or greater than (↑) statistically derived upper and lower serum thyroid concentrations.
(DOC)
