# Correction: Dietary Iodine Sufficiency and Moderate Insufficiency in the Lactating Mother and Nursing Infant: A Computational Perspective

**DOI:** 10.1371/journal.pone.0155169

**Published:** 2016-05-03

**Authors:** The PLOS ONE Staff

The first author’s name is spelled incorrectly. The publisher apologizes for the error. The correct name is: Jeffrey W. Fisher. The correct citation is: Fisher JW, Wang J, George NI, Gearhart JM, McLanahan ED (2016) Dietary Iodine Sufficiency and Moderate Insufficiency in the Lactating Mother and Nursing Infant: A Computational Perspective. PLoS ONE 11(3): e0149300. doi:10.1371/journal.pone.0149300
