# Supplementary material for: Dietary Iodine Sufficiency and Moderate Insufficiency in the Lactating Mother and Nursing Infant: A Computational Perspective
Source: PLoS One. 2016 Mar 1;11(3):e0149300. doi: 10.1371/journal.pone.0149300 (PMC4773173; doi:10.1371/journal.pone.0149300)
Supplement: S1 Table — (DOC) [file pone.0149300.s001.doc]

S1 Table. Mother and Infant (birth to 90 days of age) Physiological Parameter Values and Calculations.

| **Parameter  (*Variable Name,* units)** | **Parameter Value or Calculation** | | **Reference and Derivation Notes** | |
| --- | --- | --- | --- | --- |
|  | **Mother** | **Infant** | **Mother** | **Infant** |
| Body Weight (*BW*, kg) | 68 | BW= 13.642*(1.0 - e^(-0.0555 * age months )^) + BW_0_ BW_0_=3.512 kg | [1] Mean=67.5 kg (n=39) | [2, 3] Growth for less than 11 months |
| Volumes | | | | |
| Plasma Volume (*Vplasma*, L) | Vplasma=VplasmaC* BW VplasmaC=0.044 L/kg | Vplasma=VplasmaC* BW VplasmaC=0.048 L/kg | [4] | [5] 4 United States infants age 7 to 21 days old, average BW= 3.255 kg and average plasma volume= 0.15575 L |
| Slowly Perfused Tissue  (*VS*, kg) | VS= VSC*BW- VTB-VT VSC=0.651 L/kg | Not used |  |  |
| Richly Perfused Tissue (*VR*, kg) | VR= VRC*BW –Vplasma –VMamFat –Vmamb –VMK VRC=0.269 L/kg | Not used |  |  |
| Thyroid Gland (*VT*, kg) | VT= VTtot- VTB Vtot=VtotC*BW VtotC=0.000235 kg thyroid/kg BW | VT=((0.063*months +1.3857)/1000) -VTB | [6] | [2, 3, 7] |
| Thyroid Plasma (*VTB*, L) | VTB=VTBC* VTtot VTBC=0.276 | VTB=VTBC *VT VTBC=0.153 | [6] | [8] |
| Milk Volume (*VMK*, L) | 0.369 |  | [9, 10] | -- |
| Mammary Gland (*VMammary*, kg) | 0.431 | Not used |  | -- |
| Mammary Fat (*VMamFat*, kg) | VMamFat=VMamFatC*VMammary VMammary=0.431 VMamFatC=0.35 | Not used | [11-15] | -- |
| Mammary Plasma (*VMB*, kg) | VMB=VMBC*VMammary VMammary=0.431 VMBC=0.276, set equal to VTBC | Not used | [14] | -- |
| Rest of Body Weight (*VBody*, kg) | Not used | VBody=0.92*BW-(VTB+VT) | -- |  |
| Flows | | | | |
| Caridac Output (*QC*, L/hr) | QC= QCC*BW^0.75^ QCC=15 L/hr/kg | QC=QCblood * 0.578 QCblood=Whole blood flow rate = 30, birth to 14 days of age 40.8 for 14 to 60 days of age 55.8 for 60 to 90 days of age | [16] | Converted whole blood flow to plasma flow using equation presented in McLanahan et al. [2]. Edgington et al. [17] for whole blood flow rates. Linear interpolation was used in between data points. |
| Thyroid gland plasma flow (*QT*, L/hr) | QT= QTC*QC QTC=0.016 | QT= QTC*QC QTC=0.016 | [6] | [6] |
| Mammary gland plasma flow (*QMamb*, L/hr) | QMamb= QmambC*QC QMambC=0.02661 |  | [18] |  |
| Richly Perfused plasma flow (*QR*, L/hr) | QR= QRC*QC-QMamb QRC=0.76 | Not used | Calculated | -- |
| Slowly Perfused plasma flow (*QS*, L/hr) | QS= QSC*QC-QT QSC=0.24 | Not used | Calculated | -- |
| Rest of Body plasma flow (*QBody*, L/hr) | Not used | QBody=QC-QT | -- | -- |
| Urine Production (*VUrine*, model specific) | 1.453 L/d | VUrine (L/hr)=VUrineC*BW VUrineC=0.005 (L/hr/kg) | [19] Daily urine volume in lactating women, 0.93 ml/hr/kg, (n=55). | [20] Estimated 0.005 L/hr based on 4 hr measurements, infant void volume at 3 months of age |

# S1 Table References
