# Supplementary material for: Dietary Iodine Sufficiency and Moderate Insufficiency in the Lactating Mother and Nursing Infant: A Computational Perspective
Source: PLoS One. 2016 Mar 1;11(3):e0149300. doi: 10.1371/journal.pone.0149300 (PMC4773173; doi:10.1371/journal.pone.0149300)
Supplement: S2 Table — (DOC) [file pone.0149300.s002.doc]

**S2 Table. Mother and Infant (birth to 90 days of age) Iodide Parameter Values and Calculations.**

| **Parameter  (*Variable Name,* units)** | **Parameter Value or Calculation** | | **Reference and Derivation Notes** | |
| --- | --- | --- | --- | --- |
|  | **Mother** | **Infant** | **Mother** | **Infant** |
| Thyroidal Iodide | | | | |
| Maximal thyroidal iodide pool  (CmaxthybindC, nmol) | Amaxthybind (nmole)= CmaxthybindC * VT * 1000 CmaxthybindC = 1.13x10^4^ nmol/g thyroid gland | Amaxthybind (nmole)= CmaxthybindC*VT*1000 CmaxthybindC = 1716.7 nmol/g thyroid gland | [1] targeted 15+mg for thyroidal iodide stores | [2] healthy newborns from Canada, 292 µg in thyroid gland (n=13, SEM=±47 µg). [1] Model prediction in term fetus using 250 µg/day of iodine intake for pregnant mother (0.302 mg per thyroid gland or 0.217 µg/g thyroid gland (VT=1.386 g at birth). |
| Thyroidal uptake by NIS  Maximum Velocity Vmaxthy = nmol/hr for mother and VmaxthyC = nmol/hr/kg for infant      Affinity Constant (KmNIS, nmol/L) | Vmaxthy = Vmaxthy  VmaxthyC (nmol/hr)= 95824 at postpartum day 1  70875 at postpartum day 14  46620 at postpartum day 21  38430 at postpartum day 90     3.15 x 10^4^ | Vmaxthy = VmaxthyC *BW VmaxthyC = 4763 (birth to 21 days of age, then 2060 nmol/hr/kg until 90 days         3.15 x 10^4^ | Aboul-Khair et al. [3] reported thyroidal clearance (L/hr) of systemic iodide, equal to 2.25, 1.48, and 1.22 L/hr at 2, 6, and 12 weeks postpartum. This was converted using clearance L/hr = Vmax/Km. At birth used Vmax similar to pregnant woman (Lumen et al., 2013).  [1, 4, 5] | [6] Clearance is 2.5 ml/min/kg (n=3, birth to 21 days of age) and 1.1 ml/min/kg(n=4, birth to 90 days of age),for healthy infants from Belgium, VmaxthyC_Ni = Clearance (L/hr/kg)x Km_NISi for NIS (3.15e4 nmol/L)    [1, 4, 5] |
| Organification  (Kthybind, (nmol/L*hr)^-1^) | Kthybind = KthybindC * VT KthybindC = 0.0001 (nmol/L*hr/kg thyroid weight)^-1^ | Kthybind (/nmol/L*hr)=KthybindC*VT KthybindC=0.1 /nmol/L*hr/kg thyroid weight | Fit value to predict thyroid stores near the maximal concentration for euthyroid condition | Fit, second order rate, Fisher et al., 2013, scaled to thyroid weight because of growth of the thyroid gland |
| Breast Milk and Excretion | | | | |
| Volume of Breast Milk Ingested (NURSE, L/d) | Not used | NURSE (L/d) = NURSEC*BW*1000 NURSEC (g/kg/d) = 13 for day 1   40 for day 2  98 for day 3   140 for days 4 and 5   154 for day 30 (1 month)  148 for day 60 (2 months)  146 for day 90 (3 months) |  | [7] (first 5 days after birth), n=3,10,10,11,11 for each day, respectively  [8] (1st and 2nd months post-partum), n=25 for each month Dewey et al. 1991 (3^rd^ month post-partum, n=73) |
| Urinary Excretion of Iodide  Mom: (CLurine, L/hr) Infant: (KurineC, L/hr/kg) | CLurine (L/hr) = 5 L/hr at delivery  4 L/hr up to 6 weeks postpartum  3 L/hr at 12 weeks postpartum | Kurine (L/hr) = KurineC*BW^0.75^  KurineC = 0.044 L/hr/kg birth to 12.5 days  0.050 L/hr/kg from 12.5 to 21 days  0.055 L/hr/kg from 21 days to 90 days | Lactating woman – Aboul-Khair et al. [3], at birth 4.3 L/hr and at 2, 6, and 12 weeks postpartum, 3.1, 2.2, and 2.1 L/hr, respectively. Increased values to predict central tendency for Leung et al. [9] data | [6] 0.74 ml/min/kg from birth to 3 weeks (n=3) and then after is 1.1 ml/min/kg (n=4). Table functions predictions were smoothed out by adding transitions between the two urinary excretion estimates. |
| Milk uptake by NIS Maximum Velocity (Vmaxmilk, nmol/hr)  Affinity Constant (KmNIS, nmol/L) | Vmaxmilk = VmaxmilkC * BW^0.75^ VmaxmilkC = 7000 for postpartum days 1 to 15,  with linear decrease to 6500 by postpartum day 90  3.15 x 10^4^ | Not used | Fit to iodide concentrations in breast milk from Pearce et al. [10]   [5] |  |
| Permeability Coefficients | | | | |
| Thyroid (PAthyC, L/hrkg) | PAthy= PAthyC*BW^0.75^ PAthyC= 0.00001 L/hr/kg | PAthy = PAthyC * BW^0.75^ PAthyC = 0.01 L/hr/kg | [1] decreased from 1x 10^-4^ to fit to be consistent with expected thyroidal iodide concentration stores in adults (very low concentrations) | Fit to be consistent with empirical data on thyroidal iodide stores |
| Milk (PAmamductC,L/hr/kg) | PAmamduct = PAmamductC*BW^0.75^ PAmamductC = 0.001 L/hr/kg | Not used | [5] | Value unknown, set to reflect low lipid solubility |
| Mammary Fat (PAmamfatC, L/hr/kg) | PAmamfat = PAmamfatC*BW^0.75^ PAmamfatC = 0.001 L/hr/kg |  | No values available in literature. Set to reflect low lipid solubility |  |
| Partition Coefficients (unitless) | | | | |
| Thyroid gland Pthy Slowly Perfused PS Richly Perfused PR Breast Milk Pmilk Rest of body Pbody | 0.15     0.21 | 0.15 0.18 0.4 1.0 | [1, 11] | [1]  Body/plasma value of 0.21 is intermediate between slowly perfused (0.18) and richly perfused (0.4) |

**S2 Table References**
