# Supplementary material for: Dietary Iodine Sufficiency and Moderate Insufficiency in the Lactating Mother and Nursing Infant: A Computational Perspective
Source: PLoS One. 2016 Mar 1;11(3):e0149300. doi: 10.1371/journal.pone.0149300 (PMC4773173; doi:10.1371/journal.pone.0149300)
Supplement: S3 Table — (DOC) [file pone.0149300.s003.doc]

S3 Table. Mother and Infant (birth to 90 days of age) Thyroid Hormone Parameter Values and Calculations.

| **Parameter  (*Variable Name,* units)** | **Parameter Value or Calculation** | | **Reference and Derivation Notes** | |
| --- | --- | --- | --- | --- |
|  | **Mother** | **Infant** | **Mother** | **Infant** |
| Thyroxine (T4) | | | | |
| Volume of Distribution (VDT4, L) | VDT4 = VDT4C * BW  VDT4C = 0.09 L/kg | VDT4 = VDT4C * BW VDT4C = 0.31 L/kg | fit: ≈11 % BW in Lumen et al. 2013, small decrease to fit to serum T4 levels reported in NHANES [1] for 16 lactating women | [2] 7 children up to 12 months of age from Italy were intravenously injected with radiolabeled thyroxine |
| Thyroidal Production  (KprodT4C, (hr)^-1^) | KprodT4 = KprodT4C*BW^0.75^  KprodT4C = 1.1 x 10^-6^ (hr/kg)^-1^ | KprodT4 = KprodT4C * BW KprodT4C (nmol/hr/kg) = 0.535 from birth to 4 days  0.375 from 4 to 7 days  0.322 from 7 to 90 days | fit: 2.45 x 10^‑6^, Lumen et al. 2013, small increase fit to serum T4 levels in NHANES [1] for 16 lactating women | [3] 10 ug thyroxine/kg/day from birth to 4 days of age, 7 ug/kg/day from 4 days of age to 30 days, and 6 ug/kg/day from 1-12 months of age |
| Metabolism by Deiodinase I or II, T4 🡪 T3  (KmetT4C, (hr)^-1^) | KmetT4 = KmetT4C*BW^0.75^  KmetT4C = 1.4 x 10^-4^ (hr/kg)^-1^ | KmetT4 = KmetT4C * BW KmetT4C = 0.0035 birth to 10 days  0.0023 from 10 to 90 days | [4] 1.9 x 10^-4^, slight adjustment for lactating woman | Fit to predicted plasma T4 and fT4 from Lem et al. [5] |
| Metabolism by Deiodinase III, T4 🡪 rT3 (T3frac, unitless) | 0.50 | 0.50 from birth to 5 days 0.45 from 5 to 6 days 0.40 from 6 to 30 days 0.30 from 30 to 90 days | [4] | Chopra et al. [6] estimates based on serum concentration profiles over 30 days in 18 infants from the United States. Lumen et al. [4] estimates in the term fetus. Richard et al. [7] information on ontogeny of iodothyronine deiodinases |
| Fraction of serum total T4 as free T4  (FfT4, unitless) | 1 x 10^-4^ | 1.17 x 10^-4^ | [4] 9x10^-5^, slight adjustment for lactating woman | Initial value from Gemelli et al. [8], 11 newborns from Italy with ratio of fT4/T4 = 9.6x10^-4^, fit to data from Lem et al. [5] |
| Urinary excretion of T4 (CLurineT4, L/hr) | CLurineT4 = CLurineT4C*BW^0.75^ CLurineT4C= 0.6 L/hr/kg | CLurineT4 = CLurineT4C *BW^0.75^ CLurineT4C= 0.00005 L/hr/kg | Fit to target of less than 2 µg/day of T4 in urine, 1.6 u µg/day euthyroid lactating woman. Habermann et al. [9] reported excretion of 1.4 µg/day of T4 in urine over 24 hr in 20 non-lactating individuals | [9]Targeted 0.3% of T4 is excreted as urine. |
| Fecal excretion of T4 (CLfecesT4, L/hr) | CLfecesT4 = CLfecesT4C*BW^0.75^ CLfecesT4C = 2.0 L/hr/kg | CLfecesT4 = CLfecesT4C*BW^0.75^  CLfecesT4C = 0.0002 L/hr/kg | Fit to target, 10% of T4 excreted in feces for euthyroid lactating woman. Oddie, et al. [10] reported that in nonlactating adults 15% of administered ^131^I-thyroxine was found in feces. | [10] Adult humans 10-15% of administered ^131^I-thyroxine was found in feces. Fit to a targeted 8% of T4 formed per day based on estimates for maturation from Saghir et al. [11]. |
| Milk excretion of T4  (CLmilkT4C, L/hr) | CLmilkT4 = CLmilkT4C*BW^0.75^  CLmilkT4C = 0.3 L/hr/kg | Not used | Fit to predict < 1 µg/day of T4 ingested in milk (0.6 µg/day [12]) |  |
| 3,5,3’-Triiodothyronine (T3) | | | | |
| Volume of Distribution  (VDT3, L) | VDT3 = VDT3C*BW VDT3C = 0.35 L/kg | VDT3 = VDT3C*BW VDT3C = 0.304 L/kg | Fit: 0.44, [4], slight increase for lactating woman | [4] Term fetus value |
| Thyroidal Production (KprodT3, hr^-1^) | KprodT3 = KprodT3C* BW^0.75^  KprodT3C = 4.0 x 10^-7^ (hr/kg)^-1^ | KprodT3 (hr^-1^) = KprodT4C *BW*0.091 KprodT4C as described above | [4] Assume thyroidal production of T3 is near 1/11 of T4 production rate | [4] Assume thyroidal production of T3 is 1/11 of T4 production rate |
| Metabolism by Deiodinase I and II (KmetT3, hr^-1^) | KmetT3 = KmetT3C* BW^0.75^ KmetT3C = 2.5 x 10^-3^ (hr/kg)^-1^ | KmetT3 (hr^-1^) = KmetT3C*BW KmetT3C = 0.12 L/hr/kg birth to 10 days 0.09 L/hr/kg 10 to 90 days | [4] Slight adjustment for lactating woman | Fit to describe T3 time course data from Lem et al. [5] |
| Fecal excretion of T3  (CLfecesT3, L/hr) | CLfecesT3 = CLfecesT3C* BW^0.75^ CLfecesT3C= 0.005 L/hr/kg | CLfecesT3 (L/hr) = CLfecesT3C*BW^0.75^  CLfecesT3C= 0.0009 L/hr/kg | Fit: < 8% of T3 excreted in feces, Fisher and Oddie [13] reported 10.3% of administered ^131^I-thiiodothyronine was found in feces of adults from the United States (n=18) | [13] 10.3% of administered ^131^I-thiiodothyronine was found in feces of adults from the United States (n=18), assumed that 4.5% of T3 formed per day is excreted in feces, because of immature status [11] |
| Urinary excretion of T3 (CLurineT3, L/hr) | CLurineT3 = CLurineT3C* BW^0.75^ CLurineT3C= 0.003 L/hr/kg | Not used | Fit to target < 3 µg/d excreted in urine, lactating woman was 2.3 µg/d, Habermann et al. [9] reported excretion of 1.7 µg/day of T3 in urine over 24 hr in 20 individuals |  |
| Milk excretion of T3  (CLmilkT3, L/hr/kg) | CLmilkT3 (L/hr) = CLmilkT3C* BW^0.75^ CLmilkT3C= 2 x 10^-4^ L/hr/kg | Not used | Fit to predict < 0.1 µg/day of T3 ingested in milk (excretion rate not reported, ratio of extraction rates for T4/T3 ≈7-9. |  |

**S3 Table References**
