# Supplementary material for: Dietary Iodine Sufficiency and Moderate Insufficiency in the Lactating Mother and Nursing Infant: A Computational Perspective
Source: PLoS One. 2016 Mar 1;11(3):e0149300. doi: 10.1371/journal.pone.0149300 (PMC4773173; doi:10.1371/journal.pone.0149300)
Supplement: S4 Table — Model predictions of serum thyroid hormones were in agreement, less than (↓), or greater than (↑) statistically derived upper and lower serum thyroid concentrations. (DOC) [file pone.0149300.s004.doc]

S4 Table. Moderately low maternal dietary iodine intake (50 µg/d) with predicted and observed concentrations of iodide in lactating mother and nursing infant urine and breast milk, and serum thyroid hormone concentrations in lactating mother and nursing infant. Model predictions of serum thyroid hormones were in agreement, less than (↓), or greater than (↑) statistically derived upper and lower serum thyroid concentrations.

| Reference | Tissue Analysis | Measurement | Model Prediction iodide intake 50 µg/d |
| --- | --- | --- | --- |
| [1] (Sudan), 90 days postpartum, n≈49 | Maternal Urinary Iodide   Maternal Thyroid Hormones | 50.8 µg/L, 25 and 75% =25.4 and 63.5   **fT4**= 0.0097 nmol/L, 25-75% CI = 0.0085-0.0104 nmol/L  **T3**= 2.2 nmol/L, 25-75% CI = 1.9-2.5 nmol/L | 27 µg/L    0.007 nmol/L  **↓**0.89 nmol/L |
| [2] (Denmark), 5 days postpartum, n=90 for nonsmoking mother- infant pairs | Maternal Urinary Iodide  Infant Urinary Iodide  Breast Milk Iodide   Maternal Thyroid Hormones      Infant Thyroid Hormones | 40.8 µg/L,  5-95% CI=34.6-48.1 µg/L   50.4 µg/L,  5-95% CI= 42.1-60.3 µg/L   53.8 µg/L ,  5-95% CI= 45.5-63.5 µg/L  **T4**= 178 nmol/L,  5-95% CI =171-186 nmol/L **fT4**=0.00853 nmol/L, 5-95% CI=0.00822-0.00884 nmol/L **T3**= 2.35 nmol/L,  95% CI=2.25-2.45 nmol/L  **T4**= 159 nmol/L, 5-95% CI =153-165 nmol/L **fT4**=0.01246 nmol/L, 5-95% CI=0.01206-0.01273 nmol/L **T3**= 0.84 nmol/L, 5-95% CI=0.79-0.88 nmol/L | 41 µg/L    ≈30 – 69 µg/L (trough and peak)  ≈49.1- 77.3 µg/L (trough and peak)  **↓**126 nmol/L ↑0.013 nmol/L  ↓1.3 nmol/L  ↓116 nmol/L ↑0.02 nmol/L  ↑2.0 nmol/L |
| [3] (Texas, United States), n= 31 for postpartum days 30-45 and n=17 for postpartum days 75-90 | Breast Milk Iodide Days 30-45  Days 75-90 | 47.5 ± SD= 30.7-64.2 µg/L  42.3 ± SD= 33.6-51. µg/L | ≈23-46 µg/L (trough and peak) ≈23-47 µg/L (trough and peak) |
| [4] Morraco, 121 mothers and infants, baseline data, Days 10-19 postpartum | Breast Milk Iodide  Maternal Urinary Iodide  Infant Urinary Iodide  Maternal Serum T4  Infant Serum T4 | 40.8 (IQR=26.1-86.4) µg/L  37 (IQR=22-72) µg/L   73 (IQR=26-131) µg/L   88.5 (IQR=72.0-111.5) nmol/L  120. (IQR=99.6-142.0) nmol/L | 27 -52 µg/L (trough and peak) 33 µg/L   28-68 µg/L (trough and peak)    99 nmol/L   118 nmol/L |

**S4 Table References**

1. Eltom A, Eltom M, Elnagar B, Elbagir M, Gebre-Medhin M. Changes in iodine metabolism during late pregnancy and lactation: a longitudinal study among Sudanese women. Eur J Clin Nutr. 2000;54(5):429-33.

2. Andersen SL, Nohr SB, Wu CS, Olsen J, Pedersen KM, Laurberg P. Thyroglobulin in smoking mothers and their newborns at delivery suggests autoregulation of placental iodide transport overcoming thiocyanate inhibition. Eur J Endocrinol. 2013;168(5):723-31.

3. Hannan MA, Faraji B, Tanguma J, Longoria N, Rodriguez RC. Maternal milk concentration of zinc, iron, selenium, and iodine and its relationship to dietary intakes. Biol Trace Elem Res. 2009;127(1):6-15.

4. Bouhouch RR, Bouhouch S, Cherkaoui M, Aboussad A, Stinca S, Haldimann M, et al. Direct iodine supplementation of infants versus supplementation of their breastfeeding mothers: a double-blind, randomised, placebo-controlled trial. Lancet Diabetes Endocrinol. 2014;2(3):197-209.
